# Right posterior theta reflects human parahippocampal phase resetting by salient cues during goal-directed navigation

**DOI:** 10.1162/IMAG.a.105

**Published:** 2025-09-08

**Authors:** Malte R. Güth, Andrew Reid, Yu Zhang, Sonja C. Huntgeburth, Ravi D. Mill, Alain Dagher, Kim Kerns, Clay B. Holroyd, Michael Petrides, Michael W. Cole, Travis E. Baker

**Affiliations:** Center for Molecular and Behavioral Neuroscience, Rutgers University, Newark, NJ, United States; Graduate Program in Neuroscience, Rutgers University, Newark, NJ, United States; Department of Psychology, University of Tilburg, Tilburg, Netherlands; Zhejiang Lab, Hangzhou, China; Montréal Neurological Institute, McGill University, Montréal, Québec, Canada; Department of Psychology, University of Victoria, Victoria, BC, Canada; Department of Experimental Psychology, Ghent University, Ghent, Belgium

**Keywords:** spatial navigation, parahippocampal gyrus, theta oscillations, phase resetting, simultaneous EEG-fMRI

## Abstract

Animal and computational work indicate that phase resetting of theta oscillations (4–12 Hz) in the parahippocampal gyrus (PHG) by salient events (e.g., reward, landmarks) facilitates the encoding of goal-oriented information during navigation. Although well studied in animals, this mechanism has not been empirically substantiated in humans. In the present article, we present data from two studies (Study 1: asynchronous electroencephalography (EEG)-magnetoencephalography (MEG) | Study 2: simultaneous EEG-fMRI) to investigate theta phase resetting and its relationship with PHG blood oxygenation level dependent (BOLD) activation in healthy adults (aged 18–34 years old) navigating a virtual T-maze to find rewards. In the first experiment, both EEG and MEG data revealed a burst of theta power over right-posterior scalp locations following feedback onset (termed right-posterior theta, RPT), and RPT power and measures of phase resetting were sensitive to the subject’s spatial trajectory. In Experiment 2, we used probabilistic tractography data from the human connectome project to segment the anterior and posterior PHG based on differential connectivity profiles to other brain regions. This analysis resulted in a PHG subdivision consisting of four distinct anterior and two posterior PHG clusters. Next, a series of linear mixed effects models based on simultaneous EEG-fMRI data revealed that single-trial RPT peak power significantly predicted single-trial hemodynamic responses in two clusters within the posterior PHG and one in the anterior PHG. This coupling between RPT power and PHG BOLD was exclusive to trials performed during maze navigation, and not during a similar task devoid of the spatial context of the maze. These findings highlight a role of PHG theta phase resetting for the purpose of encoding salient information during goal-directed spatial navigation. Taken together, RPT during virtual navigation integrates experimental, computational, and theoretical research of PHG function in animals with human cognitive electrophysiology studies and clinical research on memory-related disorders such as Alzheimer’s disease.

## 1. Introduction

Finding refuge, food, or mates depends on an animal’s ability to accurately encode salient events within the context of their goals during navigation. Prior theoretical and animal work indicate that the timing of neuronal activity within the hippocampus and parahippocampal gyrus (PHG; in rodents perirhinal, postrhinal, and entorhinal cortical areas; Burwell et al., 1995) with respect to the ongoing local field potential oscillating at a theta rhythm (4–12 Hz in rodents) plays a critical role in this cortical process (Hasselmo et al., 2009; Huerta & Lisman, 1995; Mizuseki et al., 2009; Newman & Hasselmo, 2014). When salient events or cues such as rewards and navigationally relevant landmarks are presented in the animal’s environment, the phase of the theta rhythm is reset (Buzsáki et al., 1979; Givens, 1996; Williams & Givens, 2003), a process that appears to align imperative information with optimal conditions for event encoding (Hasselmo, 2008; Hasselmo et al., 2009; Hyman et al., 2003; McCartney et al., 2004; Vinogradova et al., 1996). Animal and computational work indicate that event encoding critically depends on the phase, frequency, and temporal dynamics of PHG theta oscillations (Hasselmo & Stern, 2014; Hölscher et al., 1997; Newman & Hasselmo, 2014; Quirk et al., 2021). Computational models of spatial navigation make use of this mechanism to simulate PHG memory encoding and retrieval of events encountered during spatial navigation (Hasselmo, 2008; Hasselmo et al., 2009). In particular, phase resetting of PHG theta oscillations by location-specific input from place cells is thought to prevent accumulation of error, facilitate the encoding of landmarks and goal locations, and to enhance path integration during navigation (Burgess, 2008; Burgess et al., 2007; Hasselmo, 2008). Although phase resetting of PHG theta oscillations during navigation has been previously studied in animal and computational work, this mechanism remains poorly understood in the human brain because of the limitations of non-invasive neuroimaging methods.

Over the past decades, intracranial electrophysiological recordings in epilepsy patients have discovered movement-related theta oscillations in both the neocortex (Kahana et al., 1999) and hippocampus (Ekstrom et al., 2005) during immobile virtual navigation. Electroencephalography (EEG) and magnetoencephalography (MEG) studies have also identified functional parallels between theta oscillations found in rodents during active navigation and those recorded in humans (4–8 Hz) during virtual navigation using tasks involving spatial learning and spatial memory (Cornwell et al., 2008; Jacobs et al., 2006), self-initiated movement, speed, and direction (M.-H. Lin et al., 2022), processing of landmarks (Cornwell et al., 2008; Rounds et al., 2020), as well as path integration and orientation (C.-T. Lin et al., 2015). In a series of EEG studies, we demonstrated that feedback cues presented in a virtual T-maze environment elicit a burst of theta oscillations (4–12 Hz) over posterior electrodes, with larger amplitudes over right hemisphere channels (Baker & Holroyd, 2009, 2013). This right posterior theta (RPT) response was characterized by spectral power peaking in the theta band around 200 ms following feedback onset, and was consistent with a partial phase reset of the oscillation (i.e., concomitant increases in phase coherence and spectral power relative to baseline; Min et al., 2007; Yeung et al., 2004). We proposed that RPT arises from phase resetting and enhancement in power of ongoing EEG theta activity: RPT is generated when an event leads to resetting of the phase of ongoing EEG oscillations, such that peaks and troughs become aligned. Further, the latency, power, and phase angle of RPT were found to be sensitive to feedback cues received following a right turn relative to a left turn in the T-maze (Baker & Holroyd, 2013). RPT peaked earlier with greater power for right alleys, which was accompanied by higher alignment of phase angles.

EEG source localization analysis and fMRI data point to the PHG (in humans perirhinal, parahippocampal, entorhinal cortical areas (Burwell, 2000)) as a likely source of RPT (Baker et al., 2015; Baker & Holroyd, 2013). In particular, our previous fMRI study demonstrated that when comparing feedback processing in the T-maze task with the same task devoid of the spatial context (i.e., No-maze), there was an increase in the blood oxygenation level dependent (BOLD) response in the PHG, medial temporal–occipital cortex, and right precuneus, regions commonly activated during virtual navigation (Baker et al., 2015). Moreover, posterior and anterior PHG regions were more activated by feedback stimuli following right compared with left turns only when navigating in the maze. Together, this literature supports the hypothesis that the right PHG provides the neural substrate for the encoding of salient events within the larger system for navigation, that the PHG theta rhythm is anchored to salient events via phase resetting, and that this reset impacts encoding by shifting the timing of neuronal firing in relation to the phase of the theta rhythm (Aguirre et al., 1996; Baker et al., 2015; Ekstrom et al., 2003; Maguire, Frith, et al., 1998).

Based on the results of the studies described above, we proposed that reward-related stimuli in the maze induced a partial phase reset of the PHG theta rhythm with relatively lower latency, greater power, and greater phase alignment in right alleys than in left alleys in the maze, which we term here the RPT effect. However, this proposal is complicated by two factors. First, electrical fields are distorted by varying conductivities of tissues between the source and the electrode (i.e., volume conduction), which can in turn distort the timing and power of the recorded signal between experimental conditions (Nunez, 1981; Nunez et al., 1997; Rush & Driscoll, 1969). Second, while source localization analyses have shown that the PHG is a likely source of RPT, they do not allow for any conclusions about the temporal covariation between PHG activity and RPT across trials. Furthermore, as the exact relationship between the fMRI BOLD responses and underlying oscillatory activity is not yet fully understood (Ekstrom, 2010, 2021; Logothetis, 2008; Logothetis & Wandell, 2004), it remains to be explored whether the BOLD response observed in our previous fMRI study was indeed related to RPT.

To overcome these limiting factors, we first examined whether the RPT effect was reproducible using EEG and MEG data recorded from the same individuals. While EEG allows for the recording of activity from dipoles of varying orientations and depths (Ahlfors et al., 2010; Cohen & Cuffin, 1991), MEG records activity from tangentially oriented dipoles at a high signal-to-noise ratio (Baillet et al., 2001; Melcher & Cohen, 1988), with less distortion of oscillations through volume conduction than EEG (van den Broek et al., 1998). Replicating the RPT effect using MEG would help rule out potential confounding sources of the RPT effect. Second, we set out to examine the relationship between the BOLD signal and RPT power changes by simultaneously recording EEG and fMRI while human subjects engaged in both a spatial and a non-spatial navigation task to find rewards. These experiments aim to provide converging evidence for the proposal that RPT is produced by a PHG system specific to spatial navigation, thereby integrating experimental, computational, and theoretical findings from studies of animal hippocampal–parahippocampal function with those from the field of human EEG research.

## 2 Experiment 1: Asynchronous EEG-MEG

**Fig. 1.**
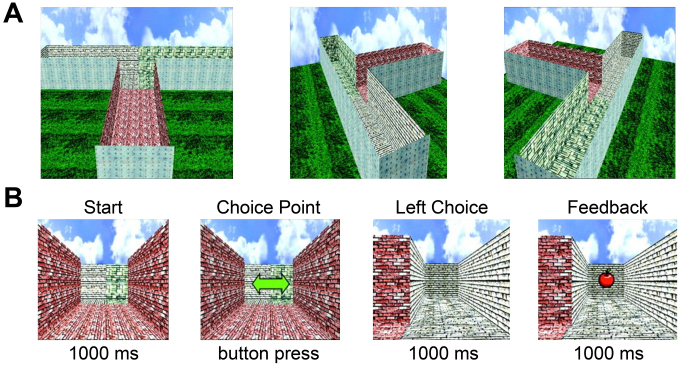
Overview of the virtual T-maze task. (A) Shape of the virtual T-maze as seen from above focused on the start position (left), the left alley (center) and the right alley (right). (B) Sequence of events comprising an example trial of the T-maze task. Top line is the event label and bottom line shows the event duration. Choice point image lasts until subject performs a button press. Behavioral analyses testing the equivalence of left and right alleys (i.e., reaction times, choice frequency) and choice patterns are provided in detail in Supplementary Figure S1.

The purpose of Experiment 1 was to use EEG (session 1) and MEG (session 2) data to test whether reward-related RPT reflects phase resetting of the ongoing theta rhythm and is sensitive to the spatial context of the feedback in the T-maze (i.e., replicate the findings from Baker and Holroyd (2013) using EEG and MEG data collected from the same individuals). For an overview of methods and results from our previous studies on RPT using the T-maze task, see Supplementary Table S7. In the virtual T-maze task (Fig. 1), subjects (N = 11; M_Age_ = 25 ± 2.9 years; 5 female) navigated toward a left or right alley in a T-maze and were asked to maximize the amount of money earned by finding as many apples (5 cents per apple) as possible. Based on our previous findings, we predicted that (i) time–frequency analysis should reveal similar feedback-related RPT and phase resetting dynamics across both EEG and MEG datasets and (ii) the RPT power, RPT latency, and phase resetting should be sensitive to the spatial location of the feedback received. As a partial phase reset is an abrupt shift in the ongoing phase of an oscillation, phase angles at the timing of RPT should align and increase in consistency across trials, as indexed by inter-trial coherence (ITC) and an analysis of the variability of phase angles.

### 2.1 Methods

#### 2.1.1 Participants

Twelve participants (M_Age_ = 25 ± 2.76 years; 5 female) participated in Experiment 1. One subject was excluded due to excessive artifacts during the MEG recordings (M_Age_ = 25 ± 2.9 years; 5 female). All participants had normal or corrected-to-normal vision and none reported a history of head injuries. Participants were graduate students recruited from the University of Victoria (BC, Canada) and were compensated C$50 for completing the study, plus a monetary bonus associated with the experimental task. Participants also received a monetary bonus equivalent to the number of rewards they found in the T-maze task. Written informed consent was obtained from all participants. The study was approved by the University of Victoria research ethics committee and was conducted in accordance with the ethical standards described in the 1964 Declaration of Helsinki.

#### 2.1.2 Virtual T-maze task

For the EEG and MEG experiments, subjects performed the virtual T-maze task (Fig. 1) which had also been used in our previous EEG studies (Baker & Holroyd, 2009, 2013; Baker et al., 2020). In this task, subjects were instructed to maximize their rewards (five cents) by going either the left or right in the virtual T-maze, which consisted of still images depicting cardinal locations in the maze. On each trial, subjects started out in the stem of the maze looking down toward a junction point where two alleys branch off orthogonally from the stem. After 1000 ms, subjects were presented with a green double arrow prompting them to choose the left (button 1, left index finger) or right alley (button 2, right index finger). As soon as a valid response was given, they moved to the junction point looking down the right or the left alley for 1000 ms and received feedback to their choice for another 1000 ms (a still image of an apple for reward or a still image of an orange for no reward). Unbeknownst to the participants, the type of feedback was selected at random (50% probability). The complete task consisted of 4 blocks with 100 trials each. At the end of the task, subjects received the total amount of rewards as a monetary bonus.

In the behavioral domain, the sum of choices (left vs. right), reaction times, and response strategies were analyzed to (a) ensure that there were no differences in how subjects responded during left and right trials and (b) to test whether subjects responded to feedback in a similar manner as in previous studies. The full results of the behavioral analysis are detailed in Supplementary Figure S1.

#### 2.1.3 EEG and MEG data acquisition

The EEG data were recorded using a montage of 63 electrode sites in accordance with the extended international 10–20 system (Jasper, 1958). Signals were acquired using Ag/AgCl ring electrodes mounted in a nylon electrode cap with an abrasive, conductive gel (Falk Minow Services, Herrsching). They were amplified by low-noise electrode differential amplifiers with a frequency response of DC 0.017–67.5 Hz (90 dB octave roll off) and digitized at a rate of 250 samples per second. Digitized signals were recorded to disk using Brain Vision Recorder software (Brain Products GmbH, Gilching, Germany). Inter-electrode impedances were maintained below 10 kΩ. Two electrodes were also placed on the left and right mastoids. The EEG was recorded using the average reference. The electrooculogram (EOG) was recorded for the purpose of ocular correction; horizontal EOG was recorded from the external canthi of both eyes, and vertical EOG was recorded from the suborbit of the right eye and electrode channel Fp2.

MEG data were acquired using a 151-channel Canadian Thin Films (CTF MEG, Coquitlam, BC, Canada) whole-head magnetometer with axial first order gradiometers (VSM MedTech). Four EEG (Cz, Fz, left mastoid, right mastoid) and two EOG channels were added, one horizontal EOG channel on the external canthi and one vertical EOG channel in the suborbit of the right eye. Participants sat in an upright position while performing their task, which was projected onto a screen in front of them. Data were recorded continuously for approximately 15 minutes and digitized at 600 Hz. To ensure a standardized co-registration of sensors and the head, fiducial head positioning coils were arranged around the nasion and the bilateral preauricular points before the experiment.

#### 2.1.4 EEG and MEG data preprocessing

Both EEG and MEG analyses were performed in Python 3.9 and MNE-python (Version 1.0.0) for M/EEG analysis (Gramfort et al., 2014). Data were bandpass filtered (low-pass: 60 Hz, high-pass: 0.1 Hz) to remove power line noise, other high-frequency noise and slow, movement induced signals. To remove ocular and movement artifacts, data were entered into an Independent Component Analysis (ICA) and decomposed into independent components (IC) using the Infomax algorithm. The ICA was run on a copy of the data, which was high-pass filtered at 1 Hz to prevent distortion through high-amplitude, low-frequency signals (Winkler et al., 2015). Next, filtered EOG signals were segmented around likely saccades and blinks. For the MEG, segments around likely heartbeat artifacts marked by their sharp, large peaks surrounded by ripples mirroring the QRS-complex were extracted as well. The time courses of each component were correlated to these ocular and cardiac artifact segments in order to identify components representing artifact signals. Furthermore, potential artifact components were inspected based on their topography and spectral composition. Components marked as artifacts were excluded from back projection to continuous data. The cleaned EEG data were re-referenced to the bilateral mastoids. Lastly, all data were segmented around feedback presentation (±2500 ms). Segments exceeding a maximum peak-to-peak amplitude of 100 μV were marked and excluded from the time–frequency analysis. For the MEG, the peak-to-peak amplitude threshold for magnetometers was set to 2000 fT.

#### 2.1.5 EEG and MEG time–frequency analysis

For the time–frequency analysis of all EEG and MEG data, cleaned segments time-locked to feedback presentation (±2500 ms) and separated by maze context and alley were exported to MATLAB (release 2020b, Mathworks, Massachusetts, USA) and analyzed using custom MATLAB scripts from our previous study (Baker & Holroyd, 2013). The frequencies ranging from 1 to 50 Hz were analyzed using a complex seven-cycle Morlet wavelet for convolution. Total spectral power was obtained by averaging the EEG spectrum across all trials and time for each subject. Time–frequency analysis on the single-trial EEG data thus yielded total theta power, including theta power that was both phase consistent (evoked) and phase inconsistent (induced) across trials with respect to the eliciting event. Evoked theta power was determined directly from the averaged Event-Related Potential (ERP), and induced theta power was then identified by subtracting the evoked theta power from the total theta power (for more details, see Baker & Holroyd, 2013; Hajihosseini & Holroyd, 2013). The relative change in the power for each condition was determined by averaging the baseline activity (-200 to -100 ms prestimulus) across time for each frequency and then subtracting the average from each data point following stimulus presentation for the corresponding frequency. This value was then divided by the baseline activity to normalize the change of power to the baseline activity. To note, spectral power was reported here unitless because it is calculated as a proportional increase/decrease relative to baseline. For each subject and feedback location, we identified the maximal increase in power for each subject’s peak theta frequency within the range from 4 to 12 Hz. The peak power and latency of each frequency band were obtained by detecting the maximum power within a 600 ms window following the onset of the feedback stimulus. This was done separately for right and left alleys. Differences between left and right alleys across subjects were assessed using Wilcoxon signed-rank tests.

#### 2.1.6 EEG and MEG intertrial coherence and phase analysis

The analysis of phase resetting relied on two main parameters: (1) ITC as a measure of correlation between phase angles across individual trials and (2) the mean phase degree angle and spread of phase. To establish phase resetting, phase coherence for the theta band after feedback across trials should increase across trials compared with baseline. Then, to determine whether this coherence is indicative of a reset, the spread of phase angles should decrease due to phase alignment. This analysis was performed on the same 5 seconds of time-domain data centered around feedback presentation (±2500 ms) as used for the time–frequency analysis. To match our previous analysis (Baker & Holroyd, 2013), the segments were trimmed to a time window from 500 ms pre-feedback to 1000 ms post-feedback. For the ITC analysis, we identified the frequency with maximum power in the EEG and MEG data for this time window. Then the algorithm implemented in the pop_newtimef function in EEGLAB (v2022, Delorme & Makeig, 2004; Makeig et al., 2002) assigns to that frequency a value between 0 and 1, where 1 indicates perfect EEG phase consistency across trials and 0 indicates random EEG phase distribution across trials. Lastly, the peak ITC value and latency were extracted within the general time window of the RPT effect. For the phase analysis, phases (in degrees) at frequencies in multiples of 2 Hz (i.e., 5–6, 7–8, 9–10, and 11–12 Hz) were extracted in a 10 ms window centered around the mean peak timing of the RPT effect averaged across alleys (EEG: 186 ms, MEG: 196 ms). In addition, the baseline window (i.e., -186 ms, -196 ms) was also analyzed as a control time period. Phase data were extracted for each trial, alley, and subject at PO8 for the EEG, as well as for each subject’s peak channel from the right parietooccipital MEG sensor cluster identified in the time–frequency analysis. Differences in ITC latency and strength between left and right alleys were tested using one-sided Wilcoxon signed-rank tests.

In order to determine the circular mean of phase angles across trials, alleys, and subjects, the circular grand mean (CGM) and the resultant vector length (RVL) were examined, using the Circstat MATLAB toolbox (Berens, 2009). These two measures served to assess differences in mean angle as well as spread of phase angles. The CGM represents an arithmetic mean for a circular scale and, thus, provides information on which phase angle, on average, occurred across trials and subjects for a particular frequency. How strongly individual phase angles spread around this mean orientation is captured by the RVL, where a value close to 0 indicates no alignment and a value close to 1 indicates ideal alignment around the mean phase direction. Hence, the RVL was used as the main indicator of phase alignment. In order to test differences in the CGM between conditions, the Harrison–Kanji test (Harrison & Kanji, 1988) and the Watson–Williams test (G. S. Watson & Williams, 1956) were employed. These tests are circular analogues of a two-factor and a one-factor ANOVA, respectively. The one-factor variant was used to determine whether there was any difference between the pre- and post-feedback window. The two-factor test assessed the effects of the factors Feedback Alley Type (left vs. right alley) and Frequency (8 Hz vs. 10 Hz) as well as their interaction. For the RVL, an analogous conventional two-way repeated measures ANOVA was used. This analysis was run using the same custom MATLAB and EEGLAB scripts as in Baker and Holroyd (2013). Post hoc tests between factor levels were performed using one-sided Wilcoxon signed-rank tests.

### 2.2 Results

#### 2.2.1 The RPT effect is observable in EEG and MEG

**Fig. 2.**
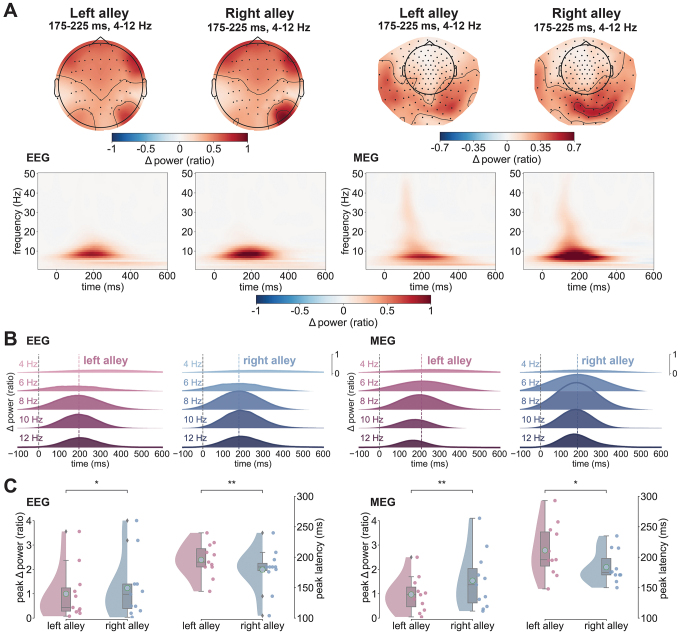
RPT effect in EEG and MEG. (A) Spectrograms with evoked power change relative to baseline (expressed as ratio) from -100 to 600 ms post-feedback separated by alleys (left vs. right alley) and modality (EEG and MEG). Spectral power is unitless because it is calculated as a proportional increase/decrease relative to baseline. Topographies at the top depict the spatial distribution of theta power from 175 to 225 ms averaged across all subjects. The left side of the panel contains the results for the EEG and the right side for the MEG. (B) Ridge plots depicting average power change for individual frequency bands (1–4, 5–6, 7–8, 9–10, and 11–12 Hz) between -100 and 600 ms post-feedback separated by left (in pink) and right alleys (in blue) and modality (EEG left side, MEG right side). Dotted colored lines mark the average peak latency across subjects for the respective alley and modality. (C) Raincloud plots showing the distributions of theta power peaks and theta power peak latencies by alley (left in pink, right in blue) and modality (EEG on the left, MEG on the right). Cyan dots mark the mean value of each distribution and gray diamonds mark outlier values. Red and blue scatterplot dots represent individual subject values. *p < 0.05, **p < 0.01.

Figure 2 illustrates the results of the time–frequency analysis of the EEG and MEG response to feedback encountered in the left and right alleys (EEG left panels, MEG right panels). Visual inspection of Figure 2A and B reveals that for both EEG and MEG data, there was a clear enhancement of theta power (4–12 Hz) peaking approximately 100–250 ms following the onset of the feedback stimulus in both the left and right alleys. This increase in power exhibited a right posterior scalp distribution with a maximum at channel location PO8 for EEG, and right parietotemporal sensor (MRO33) for MEG (see Supplementary Fig. S2A). Regarding EEG activity recorded at PO8, RPT power peaked earlier for feedback found in the right alley (M = 179.63 ms, SD = ± 33.62 ms) compared with feedback found in the left alley (M = 195.64 ms, SD = ± 26.28 ms), *W* = 0, *p* = 0.007, *d* = 0.51 (Fig. 2C), replicating our previous findings (Baker & Holroyd, 2013). This RPT latency effect of approximately 16 ms (SD = ± 17.48 ms) was not observed at the contralateral electrode channel (PO7), *W* = 27.5, *p* = 1, *d* = 0.03, indicating an asymmetric spatial sensitivity of the right hemisphere. With regard to peak power, RPT power was larger for feedback found in the right alley (M = 1.24; SD = ± 1.21) than that in the left alley (M = 1; SD = ± 1.03), *W* = 2, *p* = 0.015, *d* = 0.2, consistent with previous findings (Baker & Holroyd, 2013). Again, this effect was not observed at the contralateral electrode channel (PO7), *W* = 17, *p* = 0.285, *d* = 0.08 (see Supplementary Fig. S2).

A comparable analysis on MEG recordings revealed an elevated RPT response extending from medial occipital to right parietotemporal sensors, peaking on average around 196 ms (SD = 28.59 ms; Fig. 2A, B). For the MEG data, we identified the channels of interest by identifying the maximal peak power following the onset of feedback at each channel (averaged across conditions) and frequency. This analysis revealed a cohesive cluster of right parietooccipital sensors displaying the largest increase in theta power following feedback onset, which were pooled together for all subsequent analysis (Fig. 2A, B, right panels). Consistent with the EEG data, RPT power peaked earlier for feedback found in the right alley (M = 183.72 ms, SD = ± 25.19) than that in the left alley (M = 211.27 ms, SD = ± 40.93 ms), *W* = 8.5, *p* = 0.029, *d* = 0.79 (Fig. 2C). Further, RPT exhibited a greater increase in power for right alleys (M = 1.54; SD = ± 1.14) than for left alleys (M = 0.97; SD = ± 0.69), *W* = 4, *p* = 0.009, *d* = 0.57. Underscoring the connection between the RPT effect found in EEG and these MEG results was a significant Pearson correlation between RPT amplitude for right alleys across subjects (*r*(10) = 0.616, *p* = 0.044).

#### 2.2.2 Direction modulates phase coherence enhancement

**Fig. 3.**
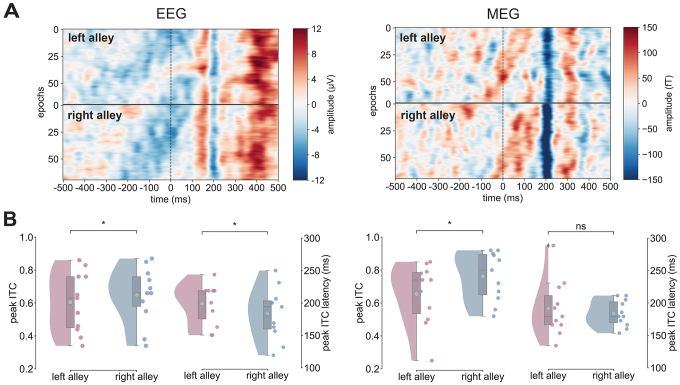
ITC magnitude and latency in EEG and MEG vary by direction. (A) First 70 phase-ordered time-domain trials of an example subject separated by modality (EEG on the left, MEG on the right) and alley (left in the top, right in the bottom) time locked to feedback presentation. Color coding represents amplitude (i.e., µV and fT) across time (-500 to 500 ms). (B) Peak ITC values and peak timings (EEG left two panels, MEG right two panels) with individual subject values plotted as colored dots separated by alley (left vs. right). ns: not significant; *p < 0.05.

Figure 3A presents an example of the single-trial EEG epochs associated with feedback onset, recorded at EEG channel PO8 (Fig. 3A, left panel, EEG) and the right parietooccipital MEG sensor MRO21 (Fig. 3A, right panel, MEG) for a single subject. The data are sorted by phase (−π to π, top to bottom) relative to feedback onset for feedback stimuli encountered in the left (top) and right (bottom) alleys. Because we had previously identified RPT bands around 8 and 10 Hz (2 Hz bandwidth; 7–10 Hz) as the frequencies with the strongest phase consistency and highest sensitivity to spatial context (left vs. right alley) (Baker & Holroyd, 2013), all subsequent analyses focused on these high theta frequency bands. Note that the EEG and MEG data are color coded such that extreme negative voltages are plotted in blue and extreme positive voltages are plotted in red. Visual inspection of Figure 3A shows strong phase consistency across trials, an impression confirmed by an analysis of ITC values, a measure of the degree to which phases of a particular frequency range synchronize across trials. Strong ITC is consistent with Makeig et al. (2002) but not diagnostic of Yeung et al. (2004, 2007) phase resetting of EEG oscillations at that frequency band, since strong ITC can occur without a reset.

Regarding EEG recordings, an analysis of phase coherence on the group level in the high theta band (7–10 Hz) revealed a large increase in ITC (M = 0.63 μV, SD = 0.16 μV) peaking around 191 ms (SD = 32.96 ms) following feedback onset. This ITC peak increase was significantly larger for right alleys (M = 0.65 μV, SD = 0.16 μV) than for left alleys (M = 0.61 μV, SD = 0.17 μV), *W* = 12, *p* = 0.031, *d* = 0.24. In addition, the peak timing of ITC was significantly earlier for right alleys (M = 183.84 ms, SD = 38.18 ms) than for left alleys (M = 199.11 ms, SD = 30.33 ms), *W* = 49, *p* = 0.014, *d* = 0.42 (Fig. 3B). Both results are a replication of our precious findings (Baker & Holroyd, 2013). With regard to the MEG data, an analysis of phase coherence in the high theta band (7–10 Hz) also revealed a large increase in ITC (M = 0.71 fT, SD = 0.15 fT) peaking around 188 ms (SD = 22.38 ms) following feedback onset. The peak ITC value and their latencies exhibited the same pattern of results as in the EEG. Right alleys were marked by a significantly larger ITC peak (M = 0.76 fT, SD = 0.14 fT) than left alleys (M = 0.66 fT, SD = 0.18 fT; *W* = 9, *p* = 0.016, *d* = 0.64), but there were no differences between left (M = 191.52 ms, SD = 41.1 ms) and right alleys (M = 188.92 ms, SD = 19.41 ms) in terms of ITC peak latencies, *W* = 28, *p* = 0.52, *d* = 0.23.

#### 2.2.3 Salient events align EEG and MEG phases across trials

**Fig. 4.**
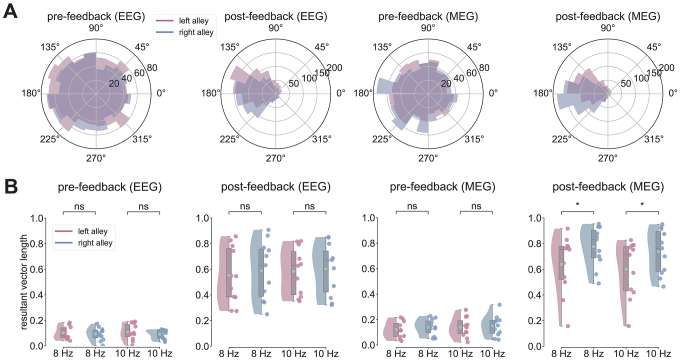
Spatial modulation of phase alignment in EEG and MEG. (A) Circular distributions of phase angles pre-feedback and post-feedback separated by method (EEG on the left, MEG on the right) and alley across subjects. Bar size corresponds to the number of trials a particular phase angle was found at the peak timing of the average ERP in the EEG and MEG. All phase values in each subject’s distribution were centered around the CGM for each condition (left: 160°/163°, right: 180°/183°). (B) Mean resultant vector pre- and post-feedback (EEG left two panels, MEG right two panels) with individual subject values plotted as colored dots separated by alley (left vs. right alley) and frequency (8 Hz vs. 10 Hz). ns: not significant; *p < 0.05.

Figure 4A shows circular histograms of the phase distribution for the frequency band associated with maximal EEG-related (left panels) and MEG-related (right panels) RPT power relative to pre- and post-stimulus onset for all subjects. Figure 4B shows the RVL of phase angles across subjects for the right (red) and left (blue) alleys recorded at channel PO8 for EEG and right parietotemporal sensor for MEG. Following the ITC analysis, the phase analysis was focused on high theta frequency bands (7–10 Hz). To test whether the spread of phase angles and thus the degree of phase alignment was different between pre- and post-feedback windows, an initial Wilcoxon signed-rank test was run to compare the RVL mean during the pre-feedback window to the post-feedback window. This was then followed by a two-factor Harrison–Kanji test on the CGM values with Feedback Alley Type (left vs. right alley), and Frequency (8 Hz vs. 10 Hz bands) as factors. For the RVL, the same factor model was implemented a standard two-way repeated measures ANOVA.

When comparing the pre- to post-feedback (±188 ms) histogram for a single example subject’s EEG recording (Fig. 4A, left panels), there was a clear alignment of phase angle distributions (see Fig. 4B, left panels), with an average CGM aligning at 141° (SD = 35.52°) and an average RVL of 0.58 (SD = 0.19) during the post-feedback window. In the pre-feedback phase alignment, RVL was significantly lower (M = 0.1, SD = 0.03; Fig. 4B, left panels) compared with the post-feedback window, *W* = 0, *p* = 0.001, *d* = 3.38. Next, an analysis between left and right alleys in the post-feedback window revealed that right alleys yielded higher RVL values and lower CGM values (8 Hz: M_CGM_ = 180°, SD_CGM_ = 55°, M_RVL_ = 0.59, SD_RVL_ = 0.22; 10 Hz: M_CGM_ = 122°, SD_CGM_ = 72°, M_RVL_ = 0.6, SD_RVL_ = 0.19) than left alleys (8 Hz: M_CGM_ = 160°, SD_CGM_ = 54°, M_RVL_ = 0.55, SD_RVL_ = 0.21; 10 Hz: M_CGM_ = 100°, SD_CGM_ = 77°, M_RVL_ = 0.58, SD_RVL_ = 0.18), but no main effects nor interactions were observed for either measure (*p* > 0.05).

With regard to MEG recordings, the histogram of phase angles at approximately 196 ms (Fig. 4A, right panels) shows a significant alignment of phase angles from pre- (M_CGM_ = 187°, SD_CGM_ = 39°, M_RVL_ = 0.14, SD_RVL_ = 0.05) to post-feedback (M_CGM_ = 173°, SD_CGM_ = 71°, M_RVL_ = 0.69, SD_RVL_ = 0.17), *W* = 0, *p* = 0.003, *d* = 4.17. When comparing left and right alleys in the post-feedback window, the two-way repeated measures ANOVA for RVL data revealed a significant main effect of Feedback Alley Type (*F*(1, 10) = 7.66, *p* = 0.019, $\eta_{p}^{2}$ = 0.43) such that right alleys displayed the highest concentration of the phase data around the mean direction (8 Hz: M_CGM_ = 163°, SD_CGM_ = 110°, M_RVL_ = 0.78, SD_RVL_ = 0.16; 10 Hz: M_CGM_ = 147°, SD_CGM_ = 113°, M_RVL_ = 0.74, SD_RVL_ = 0.14) compared with that of left alleys (8 Hz: M_CGM_ = 183°, SD_CGM_ = 116°, M_RVL_ = 0.64, SD_RVL_ = 0.23; 10 Hz: M_CGM_ = 197°, SD_CGM_ = 119°, M_RVL_ = 0.6, SD_RVL_ = 0.22). There was also a significant main effect for frequency (*F*(1, 10) = 12.51, *p* = 0.005, $\eta_{p}^{2}$ = 0.56), with 8 Hz RPT being marked by significantly greater phase alignment than 10 Hz RPT regardless of Feedback Alley Type (left vs. right alley; Fig. 4B, right panels). An interaction was not observed (*F*(1, 10) = 0.008, *p* = 0.928, $\eta_{p}^{2}$ = 0.001), indicating that the difference between right and left alleys was uniform across 8 and 10 Hz. To note, no main effects nor interactions were found when comparing EEG and MEG post-feedback when using CGM as the dependent variable.

### 2.3 Discussion—Experiment 1

We recently proposed that RPT elicited by reward feedback received during virtual navigation reflects phase resetting of the ongoing theta rhythm in the right PHG, and that the RPT phase shift between feedback in left and right alleys results from differences in the timing of theta phase reset (Baker & Holroyd, 2009, 2013). Here we replicated this RPT effect using both EEG and MEG data recorded from the same group of individuals performing the T-maze task: RPT was stronger and peaked earlier when the feedback-related stimuli were presented in the right alley compared with the left alley. This effect was only observed in sensors over the right hemisphere. Consistent with the partial phase reset hypothesis, ITC analysis in the high theta frequency range (7–10 Hz) yielded a large increase in ITC at the peak timing of RPT in both EEG and MEG datasets, indicating elevated phase consistency across trials. Larger ITC peaks were found for feedback presented in the right alley than that in the left alleys in both EEG and MEG datasets, and more so EEG-related ITC values peaked earlier for right alleys than for left alleys. Moreover, a phase analysis revealed that there was a significant increase in phase alignment at the peak RPT latency compared with baseline for EEG and MEG data, and further, phase alignment was significantly larger for right alleys than for left alleys in the MEG data, replicating our previous EEG findings (Baker & Holroyd, 2013). Note that we did not replicate the phase alignment difference, as indexed by the RVL, between left and right alleys in the EEG data, possibly due to the small sample size. Together, these EEG and MEG results demonstrate that the RPT occurs as a stimulus-induced increase in power and phase consistency of theta oscillations, the timing (and phase angle) of which depends on the spatial context in which the feedback stimuli are presented (left vs. right alleys).

## 3 Experiment 2: Simultaneous EEG-fMRI

**Fig. 5.**
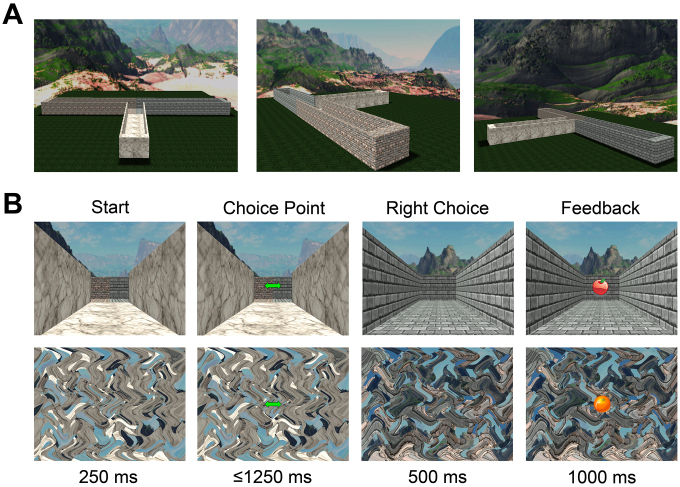
Overview of the Maze/No-maze task. (A) Shape of the virtual T-maze as seen from above focused on the start position (left), the left alley (center) and the right alley (right). (B) Example trial for Maze–No-maze task used in Experiment 2 (EEG-fMRI) depicting the T-maze (top row) and No-maze condition (bottom row). Headings in the top indicate the stimulus type and the bottom line indicates each stimulus’ duration. Scrambled feedback images were identical for left and right alleys. Behavioral analyses testing the equivalence of left and right alleys (i.e., reaction times, choice frequency) as well as choice patterns are detailed in Supplementary Figure S3.

To examine the source of RPT, we recorded EEG-fMRI simultaneously from participants engaged in the Maze/No-maze task used in our previous EEG (Baker & Holroyd, 2013) and fMRI (Baker et al., 2015) experiments (Fig. 5). In the “T-maze condition,” participants were asked to find rewards by choosing between two alley options in the virtual maze. In the “No-maze condition,” the participants engaged in a task that was formally identical to the T-maze task except that the imperative and feedback stimuli were displayed against scrambled images of the maze environment, which dissociated these events from their spatial context. This manipulation isolated the contribution of the spatial environment to the RPT effect by holding constant other aspects of task performance such as the degree of motor-related activity. Without a real-life spatial environment like the T-maze, the PHG should not differentiate between feedback following left or right button presses, removing the phase difference coding for spatial location. Hence, we predicted the following: (1) the RPT response would be elicited by the feedback stimuli in both tasks, but the RPT latency effect (earlier RPT peak in right than in left alley) would occur only in the T-maze condition and not in the No-maze condition (replicating Baker & Holroyd, 2013); (2) feedback stimuli would produce a greater hemodynamic response in the right PHG and other regions associated with virtual navigation (i.e., precuneus and medial temporal cortex) when presented in the T-maze condition than presented in the No-maze condition; (3) feedback stimuli presented in the right alley would produce a greater right PHG hemodynamic response than in the left alley in the T-maze (replicating Baker et al., 2015); and (4) trial-by-trial variation in peak RPT power should be significantly coupled to trial-by-trial variation in PHG BOLD activation, and not to BOLD activation in control regions which are unrelated to spatial navigation or outside of the medial temporal cortex (for further details, see Methods section “Integrated EEG-fMRI analysis” and Results section “Single-trial right PHG activation is predicted by RPT power”).

### 3.1 Methods

#### 3.1.1 Participants

For Experiment 2, 28 right-handed undergraduate students (M_Age_ = 23.5 years, SD_Age_ = 4.24 years; 16 female) were recruited from Rutgers University-Newark and the New Jersey Institute of Technology (NJ, USA). Written informed consent was obtained from all participants. Three of these participants were excluded due to a lack of valid trials. The resulting sample had a mean age of 23.6 years (SD_Age_ = 4.31 years; 15 female). All subjects were screened with self-report questionnaires regarding demographic information, handedness (Oldfield, 1971), spatial navigation abilities (Hegarty et al., 2002), neurological and psychiatric history, their vision, and MRI permissibility (pregnancy, metal or medical implants, etc.). Only subjects who were right handed, between 18 and 55 years of age, had normal-to-corrected vision, and had no history of neurological or psychiatric disorders or medication were allowed to participate. For their participation, subjects received course credit as well as monetary compensation equivalent to the amount of rewards they earned during the task. The study was approved by the Rutgers University Ethics Committee Board and was conducted in accordance with the ethical standards described in the 1964 Declaration of Helsinki.

#### 3.1.2 Maze/No-maze task

Participants in the simultaneous EEG-fMRI experiment engaged in the same version of the Maze/No-maze task (Fig. 5) used in our previous fMRI study (Baker et al., 2015). In this task, 192 trials were divided equally across six blocks with each trial following the same progression of events as in the virtual T-maze task (Fig. 1B). However, blocks alternated between the T-maze and No-maze condition in a counterbalanced manner across subjects. In addition, rest trials displaying a fixation cross for 3000 ms were randomly dispersed across blocks (16 per block) to optimize fMRI design efficiency. In both conditions subjects performed the same T-maze task as described before, except that in the No-maze condition there was no maze background. Instead, response prompts and feedback images were shown on scrambled versions of the maze backgrounds. As a result, the No-maze condition had no spatial context. Behavioral data, demonstrating the equivalency of left and right alleys (sum of choices, reaction times, and response strategy), are described in Supplementary Figure S3.

#### 3.1.3 EEG-fMRI data acquisition

EEG data for all 256 channels were collected at a sampling rate of 1 kHz using an MR-compatible HydroCel Geodesic sensor net (Electrical Geodesics Inc., Eugene, USA). In addition to scalp electrodes, a two-lead electrocardiogram (EKG) was recorded for later artifact correction. One active EKG lead was placed on the lower end of the sternum (4^th^ intercostal space) and one to the left of the mid clavicle line (5^th^ intercostal space). The amplifier was shielded in a Field Isolation Containment System (FICS), allowing it to be placed on the ground next to the bore. All wires were carefully straightened beside the subject to prevent hazardous wire loops. The FICS was connected via a fiber optic cable to an intermediary USB clock sync interface in the control room. At the USB interface, digital inputs from the experimental computer and the MRI clock, event triggers of the Maze/No-maze task, and MRI triggers for volume acquisition were relayed to the EEG acquisition computer. This enabled high precision synchronization of EEG and MRI acquisition necessary for MR-related artifact correction. All electrodes recorded from inside the scanner were online referenced to E257, which is the geodesic electrode placement system’s equivalent to Cz in the international 10–20 system (Jasper, 1958). Conversions of electrode positions in the geodesic sensor net to the nearest equivalent in the 10–10 system were based on Luu and Ferree (2005). Data were recorded using the Net Station software (Version 5.4.2) applying an online bandpass filter excluding data above 100 Hz and below 0.001 Hz. All electrode impedances were kept below 50 kΩ.

MRI data were collected in a 3T scanner (Trio Tim, Siemens) with a 12-channel head coil at the Rutgers Brain Imaging Center. Anatomical images were acquired with a T1-MPRAGE sequence, consisting of 176 sagittal slices (1 mm isotropic voxel; TR = 2500 ms, TE = 2.52 ms, flip angle = 9°). A dual-echo gradient-echo sequence was used to assess a B0 inhomogeneity gradient field map (TE1 = 5.19 ms, TE2 = 7.65 ms, TR = 400 ms). Functional whole brain images were collected in an axial orientation by using a T2-weighted gradient echo planar imaging sequence (TR = 2000 ms, TE = 25 ms, flip angle = 90°) with an isotropic voxel size of 3.3 mm (64 x 64 matrix; 208 x 208 mm^2^ field of view). Thirty-five slices per volume were acquired in an ascending, interleaved order. Functional image acquisition sessions lasted 15 minutes, resulting in 450 volumes for each participant. Moreover, a resting state and diffusion-weighted imaging (DWI) acquisition was added after the functional task runs, lasting approximately 12 minutes each.

#### 3.1.4 EEG data preprocessing and analysis

EEG data concurrently collected with fMRI are affected by two major MRI-related artifacts: Gradient artifacts (GA) and ballistocardiac artifacts (BCA). Both GA and initial BCA correction based on principal component analysis were carried out using Brain Vision Analyzer 2.1 (Brain Products GmbH, Gilching, Germany). Subsequent preprocessing including ICA, filtering, re-referencing, and segmentation was performed using custom scripts in Python 3.9 and MNE-python (Version 1.0.0) for M/EEG analysis (Gramfort et al., 2014).

The first step in cleaning the EEG data was to subtract GAs caused by the periodically changing magnetic fields during the acquisition of functional images. For this purpose, an average artifact correction (Allen et al., 2000; Moosmann et al., 2009) was performed. An artifact template was built from the EEG activity in 25 GA windows around a given artifact (±12 GAs). This template was subtracted by moving it across the continuous signal contaminated by GAs. To account for BCAs, a peak detection was run to find R peaks in the EKG channel. Results of this detection were inspected and manually corrected if needed. Using the first three components from a principal component analysis run on the heartbeat segments centered around the R peaks in the EKG, a new BCA template was formed and subtracted from the EEG signal (Niazy et al., 2005).

Next, the EEG signals were filtered using a Butterworth filter with a bandpass of 0.1–60 Hz. To remove residual BCAs as well as ocular and movement artifacts, all channels were entered into an ICA and decomposed into independent components using the Infomax algorithm. As in Experiment 1, a copy of the data was high-pass filtered at 1 Hz before running the ICA on this copy. When unmixing the EEG signal, 60 ICA components were extracted for each subject. BCAs are primarily caused by movement of electrodes and cardiac-related activity (Allen et al., 1998). Due to the pulsatile blood flow at temporal arteries while lying in the scanner, they occur as a prototypical progression of topographies marked by reversed polarities over the left and right hemispheres and higher amplitudes at temporal electrodes (Yan et al., 2010). Another reliable indicator of BCAs is their time course correlation to the ejection phase of the cardiac cycle, as indexed by the EKG signal’s R peak, and more broadly the QRS complex, which marks a rapid depolarization of the right and left heart ventricles (Debener et al., 2008; Shams et al., 2015). BCA components were thus identified based on their topography and Pearson correlation to the EKG segments. Components reliably categorized as BCAs at the end of this validation procedure as well as components reflecting eye or muscle movement were rejected before back-projecting the ICA to the continuous EEG signal.

After data were both GA- and BCA-corrected, they were re-referenced to the average of all scalp electrodes, excluding sensor net positions located on the cheeks or the neck. The continuous data were segmented into 5-second windows around the presentation of positive and negative feedback (±2500 ms). This was done separately for both T-maze versus No-maze trials as well as feedback presented in left and right alleys. After all preprocessing was completed, 3 out of the 28 original subjects were excluded due to a lack of valid segments. For the remaining 25 subjects, the same time–frequency analysis as described for Experiment 1 was performed on the geodesic equivalents of PO7 (E107) and PO8 (E160). For each subject, 4–12 Hz power was averaged across all conditions. Then, the peak theta frequency for each subject and condition was determined.

Peak RPT power and latency values (50–350 ms) were entered as dependent variables into two separate two-way repeated measures ANOVAs. Both tested the factors Feedback Alley Type (left vs. right alley), Task Type (Maze vs. No-maze), as well as their interaction. To ensure homogeneity of variance, a Shapiro–Wilk test was run and approximate normal distribution of the dependent variable was tested using a Levene test. Post hoc multiple pairwise comparisons of main effects and simple effects of the interaction were run using paired t-tests. Corresponding *p*-values were corrected using the Bonferroni–Holm method.

#### 3.1.5 fMRI data preprocessing and analysis

MRI data were formatted according to the international Brain Imaging Data Structure (BIDS) (Gorgolewski et al., 2016). Apart from this, the preprocessing of the functional data was performed identically to the previous study (Baker et al., 2015) using custom scripts in MATLAB (release 2020b, Mathworks, Massachusetts, USA) and SPM12. Functional data were first slice timing and then motion corrected with respect to the first image. Afterward, functional images were co-registered with the structural scan and then normalized to a standard Montreal Neurological Institute (MNI) template with 12-parameter affine registration. All normalized images were smoothed with an 8-mm^3^ FWHM Gaussian kernel.

First-level analyses were performed using a general linear model (GLM) including a constant term, six motion regressors obtained from motion correction, and eight event regressors to model activation time-locked to feedback presentation as well as orientations for the T-maze and No-maze conditions. Events were divided by feedback valence and alley. These four events were further divided by separating by T-maze and No-maze conditions, resulting in four regressors for each alley and feedback. These were built identically to previous experiments using the fMRI version of the Maze/No-maze task (Baker et al., 2015). All models were run with a canonical hemodynamic response function and its temporal derivative. Before the preprocessed time series data were statistically modeled, they were high-pass filtered with a mean cutoff period of 128 seconds. To identify voxel activation related to spatial effects, all feedback presented in the T-maze condition was contrasted to feedback presented in the No-maze condition. To identify activation associated with navigating toward the left or right, directions were contrasted separately for T-maze and No-maze conditions. Finally, linear contrasts of coefficients for each participant were used to run a second level analysis by applying paired t-tests and False Discovery Rate (FDR) correction for multiple comparisons.

Next, we constructed a PHG mask consisting of 12 bilateral ROIs based on connectivity patterns found in DWI (Baker, Reid, et al., 2017) data from the Human Connectome Project (HCP; Van Essen et al., 2012). This approach was used to extract regional variations in the connectivity profile of the selected seed region with the whole brain. To note, the boundaries of the anterior PHG (aPHG) and posterior PHG (pPHG) region were based on the definition described by Huntgeburth and Petrides (2012). The tractography‐based parcellation results yielded four subregions within the right aPHG, and two subregions within the right pPHG (Fig. 8B) (see Supplementary Materials, PHG Segmentation Analysis).

Standardized beta series values were then extracted from the 12 bilateral PHG clusters (6 per hemisphere; Fig. 8B) using nilearn NiftiMasker (Abraham et al., 2014). These time series were created by splitting regressors using the BIDS application NiBetaSeries for Python (Kent & Herholz, 2019). Instead of a conventional GLM with one beta for each event regressor, beta coefficients were estimated for every single event in the design matrix. To counteract the overlap of BOLD responses on single trials, we employed the least squares separate (LSS) approach (Mumford et al., 2012; Rissman et al., 2004). LSS improves the estimation of single-trial event-related BOLD signals with short intervals between events (i.e., 3–4 seconds) by calculating one GLM for each trial with one predictor for the trial of interest and one for the combination of all the other trials.

#### 3.1.6 Integrated EEG-fMRI analysis

To test whether EEG and fMRI data were significantly coupled on a trial-by-trial level, a series of Linear Mixed Effect (LME) models were estimated, 1 for each of the 12 PHG ROIs. This was done separately for each condition (T-maze vs. No-maze) and hemisphere (left vs. right PHG) using five centered EEG regressors (delta: 1–4, theta: 5–6, 7–8, 9–10, and 11–12 Hz) and a random intercept for each subject. Each of these EEG regressors received a fixed effects estimate (regression weight) that was transformed to a standardized beta weight and tested for significance with a t-test. *p*-Values within each model were corrected for multiple comparisons using the FDR method. To note, prevailing definitions of the human theta rhythm (4–8 Hz) (Ekstrom et al., 2005) are inconsistent with the wider animal definition (4–12 Hz) (O’Keefe, 1993; O’Keefe & Recce, 1993) and are often treated as a functionally homogeneous range. This is despite considerable variability in the locus of hippocampal theta power related to memory and spatial navigation from iEEG recordings (Bohbot et al., 2017; Jacobs, 2014; Rutishauser et al., 2010; Stangl et al., 2021; Watrous et al., 2011). This is especially relevant in human spatial navigation research given that human iEEG recordings show that there are functional specializations of low (~3 Hz) and high theta (~8 Hz) in the hippocampus during spatial navigation (Goyal et al., 2020). This view is consistent with our current and our previous time–frequency and phase resetting results (Baker & Holroyd, 2013), yielding the most prominent RPT effects in the upper theta range (7–10 Hz). Therefore, we decided to split the theta range into four regressors with a bandwidth of 2 Hz each. As a control and to differentiate against slower oscillations involved in feedback processing, we also included a delta power regressor in each model. Given the intercorrelation of EEG frequency bands, we tested the potential impact of multicollinearity on the EEG regressors and found evidence of no to at most mild multicollinearity (see Supplementary Fig. S6).

The LME models tested whether centered EEG regressors derived from single-trial evoked power (mean power from 50 to 250 ms post-stimulus) could predict variation of beta series values from the PHG ROIs. Due to increased noise on a single-trial level, each trial was baseline corrected with the mean of the baseline window (-200 to -100 ms pre-stimulus) averaged across all trials. BOLD activation in the right PHG was predicted using power values recorded at the right hemispheric EEG sensor E160, and BOLD activation in the left PHG was predicted using the left hemispheric sensor E107. When averaging single-trial power estimates for the T-maze and No-maze condition across left and right alleys, these electrodes carried the most pronounced RPT power in their respective hemisphere.

Building on this PHG analysis, we conducted two control analyses. First, the same RTP power regressors as in the PHG models were used to predict single-trial BOLD activation in the precuneus (whole brain cluster from Fig. 7A) and the middle superior temporal gyrus from the Hammersmith atlas (i.e., Heschl’s Gyrus; Hammers et al., 2003). If RPT is specifically related to the PHG, neither an area related to spatial navigation like the precuneus nor an area near the PHG that is unrelated to spatial navigation like Heschl’s gyrus should have a significant relationship with RPT. Second, we tested for couplings across the whole brain by running one LME with the same five EEG regressors for each ROI in the Glasser atlas split across the left and right hemisphere (Glasser et al., 2016). These control analyses served to test whether potential couplings between RPT power and BOLD activation were specific to the PHG.

All analyses were carried out using the lme4 package (Bates et al., 2015) in the R Programming Environment (R Core Team, 2016). Since single-trial regressors reflect a direct co-variation of two continuous variables, such as EEG power and BOLD beta series values, LMEs allowed insight into trial-by-trial co-variation. LMEs also provide the advantage of modeling single-trial variation within single subjects and to group error terms more flexibly. Here, random intercepts were included to better group individual variance for each subject. This approach has previously been successfully applied in a simultaneous EEG-fMRI study linking single-trial visually evoked gamma activity to visual cortex activation (Beldzik et al., 2019).

### 3.2 Results

#### 3.2.1 Electrophysiological evidence of the RPT effect in EEG-fMRI

**Fig. 6.**
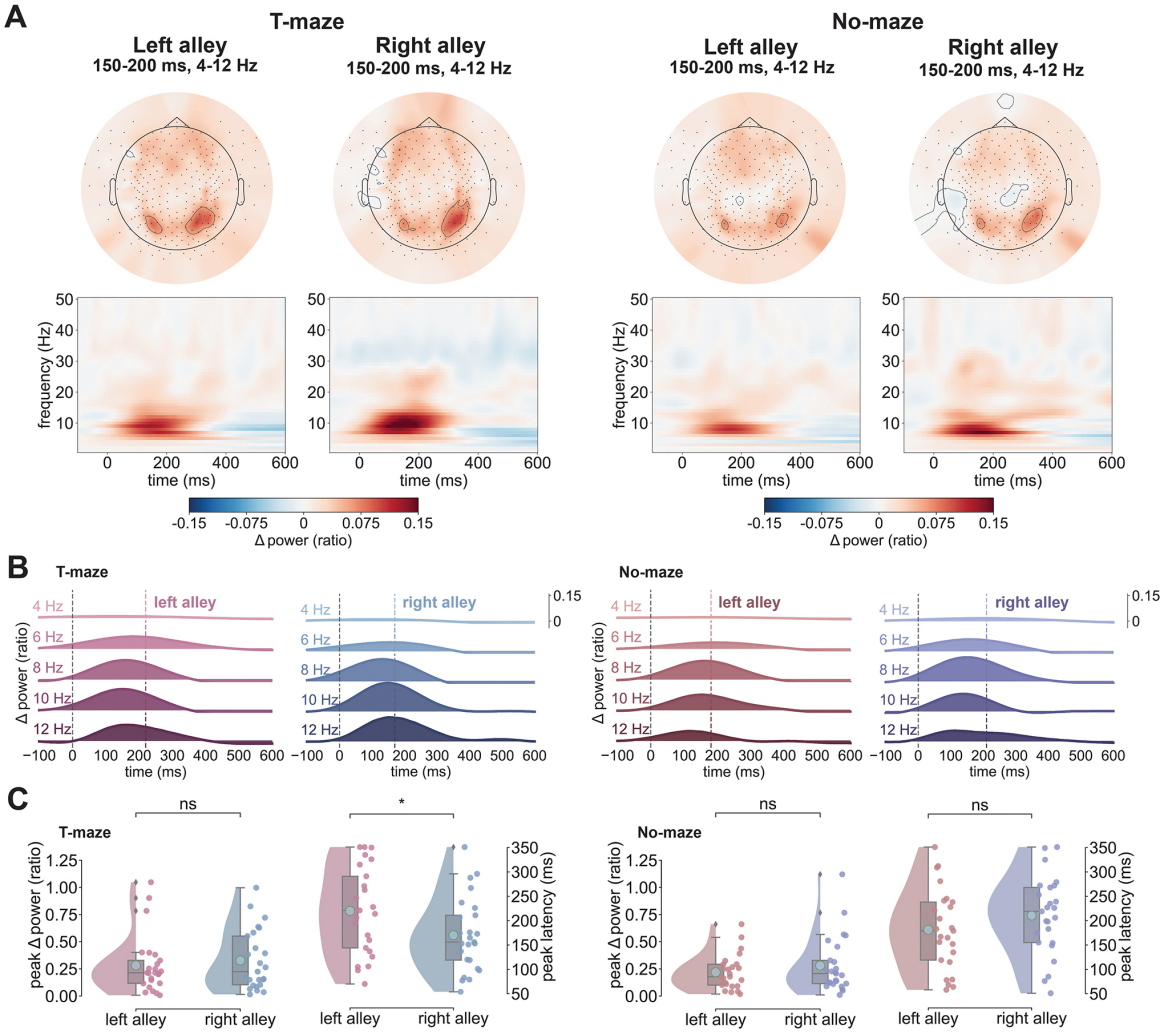
Replication of the RPT effect in simultaneous EEG-fMRI. (A) Topographies depicting the evoked power for delta (1–4 Hz) and theta bands (4–12 Hz) averaged from 150 to 200 ms post-feedback separately for Task Type (T-maze: left panels, No-maze: right panels) and alley (left alleys vs. right alleys). The spectrograms below show the corresponding evoked power from -100 to 600 ms at a right parietooccipital sensor (E160) separated by Task Type and alley. (B) Ridge plots show the same results as in A divided into the delta band (1–4 Hz) and theta bands (5–6, 7–8, 9–10, and 11–12 Hz), with the average RPT peak timing for each condition marked by a dashed colored line. Results for the T-maze condition are on the left side and those for the No-maze condition on the right side. (C) Raincloud plots depict the RPT power peaks and peak latencies for the T-maze condition on the left and those of the No-maze condition on the right, with cyan dots showing the mean value of each distribution. Alley is shown on the x-axis and colored dots represent individual subject values. ns: not significant; *p < 0.05.

Figure 6 illustrates the results of the time–frequency analysis of the electrophysiological response to feedback encountered in the left and right alley locations. Visual inspection of Figure 6A reveals a clear enhancement of theta power (between 4 and 12 Hz) peaking approximately 200 ms following the onset of the feedback stimulus in the T-maze (left) and No-maze (right) conditions. This increase in power exhibited a right posterior scalp distribution with a maximum at channels E160 and 107 of the 256-channel EEG geodesic sensor system applied in this experiment, which are equivalents of PO8 and PO7 of the standard 10–20 EEG configuration. These results are characteristic of RPT (e.g., Baker & Holroyd, 2013) and indicate that RPT can be detected when recording EEG during MRI scanning.

In line with our prediction, a repeated measures ANOVA on RPT peak latency as a function of Task (T-maze vs. No-maze) and Feedback Alley Type (left vs. right alley) revealed an interaction between Task and Feedback Alley Type, *F*(1, 24) = 10.14, *p* = 0.003, $\eta_{p}^{2}$ = 0.3 (Fig. 6B, C). A priori comparisons between alleys in the T-maze and No-maze condition indicated that for the T-maze condition, the RPT peak latency was longer for left alleys (M = 220 ms, SD = 86 ms) than for right alleys (M = 170 ms, SD = 74 ms), *t*(24) = 2.51, *p* = 0.039 (Bonferroni–Holm corrected), as we predicted. By contrast, in the No-maze condition, no difference was observed for the RPT peak latency to feedback presented following a left button press (M = 181 ms, SD = 81 ms) compared with a right button press (M = 210 ms, SD = 78 ms), *t*(24) = 1.81, *p* = 0.082. Importantly, this T-maze simple main effect was not observed at the contralateral sensor E107 (*p* > 0.05; see Supplementary Fig. S4). A comparable ANOVA on RPT power as a function of Task and Feedback Alley Type revealed a trend for a main effect of Alley Type, *F*(1, 24) = 4.067, *p* = 0.055, $\eta_{p}^{2}$ = 0.14, indicating that RPT peak values tended to be larger for right alleys (M = 0.303, SD = 0.263) than for left alleys (M = 0.25, SD = 0.217) regardless of Task Type. No other main effects nor interactions were identified.

#### 3.2.2 PHG BOLD is modulated by direction only during navigation

**Fig. 7.**
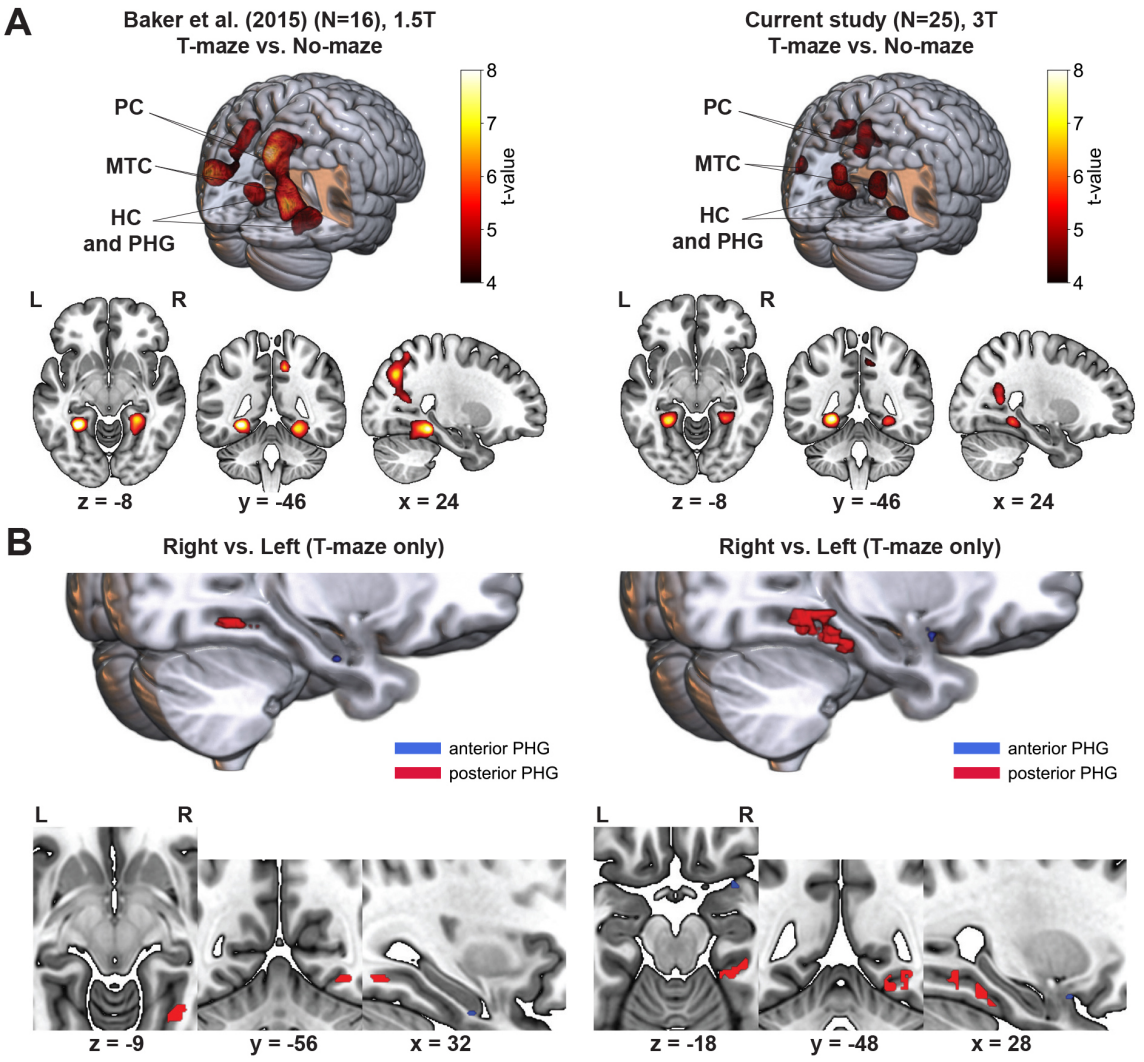
Comparison of the whole brain Maze versus No-maze contrast and ROI Right versus Left contrast results between the current and previous analysis. (A) Left side depicts the data shown in Figure 2B and C from Baker et al. (2015) (recorded using a 1.5T scanner). Plots show the results of the whole brain analysis of the maze contrast. The 3D render contains significant clusters stronger during the T-maze condition (*p* < 0.05, FDR-corrected) in (top right) and outside of the render (top left). Labels indicate the location of the clusters (PC: Precuneus; MTC: Medial Temporal Cortex; PHG: Parahippocampal Gyrus). Multiplanar slices show the color-coded t-values for the same clusters stronger for the T-maze condition in red and yellow (L/R: Left and Right). Analogous plots for the current study depicting the same T-maze versus No-maze contrast are shown on the right side. (B) Same as in A, left panels show the ROI PHG results for the T-maze versus No-maze as well as Right versus Left (Maze only) contrasts depicted in Figure 3 from Baker et al. (2015). The right side depicts the same contrasts in analogous plots for the results of the current study. 3D renders show the locations clusters specific to the T-maze condition and right alleys specifically in the T-maze condition located in the same anterior (blue; only significant on peak-voxel level) and the posterior (red; marginally significant on cluster level) PHG areas across both studies. Multiplanar slices show only the Right versus Left contrast focused on the bilateral PHG.

Figure 7A depicts the whole brain clusters for the T-maze versus No-maze contrast from Baker et al. (2015: 1.5T scanner) (left panels) and the current study (right panels: 3T scanner) that were significant after FDR correction. Consistent with our prediction and prior work, feedback stimuli presented in the virtual T-maze task relative to the No-maze task revealed significant activations in bilateral PHG (right: *t*(24) = 6.51, *p* = 0.023, FDR-corrected | left: (*t*(24) = 9.11, *p* = 0.004, FDR-corrected), left temporal-occipital cortex (*t*(24) = 6.53, *p* = 0.026, FDR-corrected), right precuneus (*t*(24) = 5.7, *p* = 0.0001, FDR-corrected), and bilaterally in the cuneus (right: *t*(24) = 8.28, *p* = 0.004, FDR-corrected; left: *t*(24) = 8.83, *p* = 0.026, FDR-corrected), brain regions commonly activated during virtual navigation tasks (Fig. 7; Table 1; Epstein, 2008; Kaplan et al., 2014; Ohnishi et al., 2006; Weniger et al., 2010). The No-maze condition displayed a significant cluster in the medial occipital gyrus (*t*(24) = 9.99, *p* = 0.0001, FDR-corrected). A complete overview of whole brain cluster statistics is given in Table 1. Next, a whole brain analysis between the hemodynamic response to feedback processing in the right alley compared with the left alley of the T-maze revealed activations largely in the motor system (e.g., left primary motor cortex to feedback encountered in the right alley, *t*(24) = 7.4, *p* < 0.0001, FDR-corrected, and activation in the right motor cortex to feedback encountered in the left alley, *t*(24) = 7.36, *p* < 0.001, FDR-corrected), as would be expected given the motor demands of the task and the results of our previous study.

**Table 1.** Whole brain cluster statistics for the T-maze versus No-maze contrast.

Cluster statistics			Peak MNI coordinates			
*p*-value (FDR-corr)	Size (voxels)	Peak t-value	x	y	z	Anatomical label
**Maze**						
0.004	471	9.11	-30	-50	-2	Left Hippocampus, Left Parahippocampal Gyrus, Left Fusiform Gyrus, Left Lingual Gyrus
0.026	222	8.83	-16	-58	16	Left Precuneus, Left Cuneus
0.004	467	8.28	20	-56	18	Left Middle Occipital Gyrus
0.026	215	6.53	-40	-78	20	Right Precuneus, Right Cuneus
0.023	261	6.51	26	-44	-8	Right Hippocampus, Right Parahippocampal Gyrus, Right Fusiform Gyrus, Right Lingual Gyrus
0.000	1194	5.70	10	-50	48	Precuneus
**No-Maze**						
0.000	4124	9.99	-6	-94	10	Cuneus

Rows show significant clusters found to be more strongly activated for the T-maze and No-maze condition, respectively.

As before, we conducted an ROI analysis focusing exclusively on the right and left PHG (Fig. 7B; left panel: results from Baker et al., 2015, right panel: current results). Consistent with our previous study, bilateral clusters were observed in a posterior region of the right PHG (*t*(24) = 6.51, *p* = 0.026, FDR-corrected) and the left PHG (*t*(24) = 8.67, *p* = 0.023, FDR-corrected) when contrasting feedback processing in the T-maze condition versus the No-maze condition. The same posterior cluster (*t*(24) = 4.4, *p_peak-level_* = 0.0001, uncorrected, *p_cluster-level_* = 0.049, uncorrected) and an anterior cluster (*t*(24) = 3.2, *p_peak-level_* = 0.002, uncorrected, *p_cluster-level_* = 0.75, uncorrected) emerged in the right hemisphere when subjects chose the right alley as opposed to left alley in the T-maze. The opposite contrast revealed no voxel values that were stronger for feedback stimuli presented in the left alley compared with that presented in the right alley of the maze, and an analysis on the left PHG ROI did not reveal any significant activation for either the left alley–right alley or right alley–left alley contrasts. These clusters directly mirrored the results from Baker et al. (2015) as can be seen when comparing the left and right panels of Figure 7B, highlighting the PHG’s robust engagement during feedback processing in a spatial environment.

#### 3.2.3 Single-trial right PHG activation is predicted by RPT power

Building on these unimodal results, the goal of the integrated EEG-fMRI analysis was to take advantage of the complementary temporal and spatial resolution of each method. By integrating the EEG and fMRI data, we hoped to determine whether or not RPT reflected a PHG theta reset mechanism for spatial navigation. It is important to note that the PHG encompasses a large portion of the medial temporal lobe, and that it has been traditionally accepted to include different regions along its anterior-posterior axis (i.e., entorhinal cortex, perirhinal cortex, and parahippocampal cortex). In addition, a significant body of research has identified several functional processes linked to the parahippocampal region (Aminoff et al., 2013; Epstein, 2008; Maguire, Frith, et al., 1998; Owen et al., 1996; Weniger & Irle, 2006). Furthermore, given the highly functional and interconnected nature of the parahippocampal region, it has recently been argued that its anatomically defined subregions should be further subdivided by its connectivity with other cortical and subcortical areas (Syversen et al., 2021). This was motivated by the conflicting results stemming from cross-species comparisons between humans, rodents, and non-human primates, as well as from human neuroimaging data on spatial memory processing, and from observations of neurodegenerative structural changes of human PHG divisions based on animal cytoarchitecture and landmarks (Syversen et al., 2021). It is likely that such conflicting results arise from variable definitions of parahippocampal subregions across studies. We, therefore, proposed that a well-defined structural connectivity-based segmentation of the human PHG was a necessary precursor for understanding how spatial contextual information is processed by the PHG during goal-directed navigation and, further, provide a more precise localization of RPT activity (Baker, Reid, et al., 2017). Thus, redefining the conventional PHG ROI using formulating criteria based both on anatomical landmarks and connectivity profiles was relevant for the interpretation of the current EEG-fMRI integration analysis, as well as for future studies examining the functional relevance of its subregions in goal-directed behavior.

**Fig. 8.**
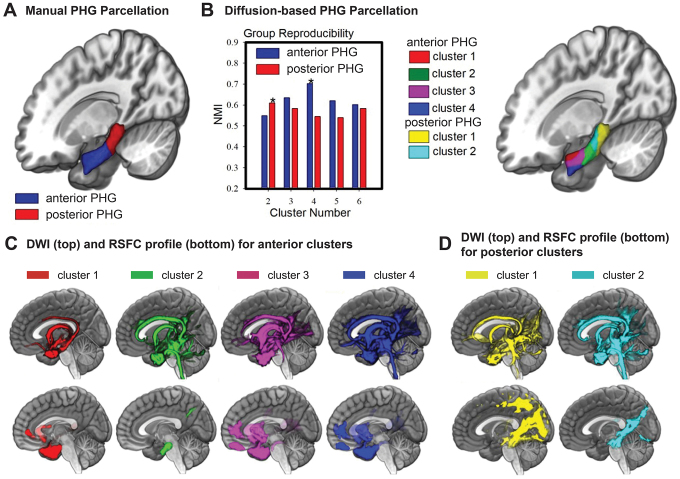
Structural and functional connectivity profile of the PHG. (A) Illustration of the PHG manual parcellation map of the anterior (in blue) and posterior (in red) PHG as established in the present article. (B) Anterior and posterior PHG diffusion parcellation results for the HCP datasets. X-axis shows size of cluster solution (i.e., two- to six-cluster solution). Normalized mutual information provides an indication of clustering consistency in which high values indicate good consistency (left panel). The figure shows that the four-cluster solution had the highest normalized mutual information value for the anterior PHG, and two-cluster solution for the posterior PHG (marked by stars). Surface view of the PHG parcellation results (right panel). (C) Population maps of the probabilistic tractography (top row) and RSFC (bottom row) results for each right anterior cluster. Statistical parametric maps are displayed using a statistic thresholded at *p* < 0.001 (FDR-corrected) with the intensity scales representing a T-value of 18. All figure maps are shown on the ICBM152 template in MNI space with MRIcroGL. (D) Population maps analogous to C for the posterior clusters.

Here, we used probabilistic tractography data from the HCP to segment the anterior PHG (aPHG) and posterior PHG (pPHG) based on differential connectivity profiles to the whole brain region (Baker, Reid, et al., 2017). A detailed description of the tractography‐based PHG parcellation analysis and statistics can be found in the Supplementary Material (PHG segmentation analysis: Diffusion-weighted and resting-state functional MRI). In brief, the boundaries of the aPHG and pPHG region were first defined by reference to the morphology of the sulci of the collateral sulcal complex as described by Huntgeburth and Petrides (2012) (Fig. 8A). Once defined, the aPHG and pPHG masks were linearly registered to native diffusion space and we then applied a data-driven connectivity-based brain parcellation procedure (i.e., spectral clustering) described in Zhang et al. (2017) and Fan et al. (2016). This method delineates the boundary of subdivisions based on the whole brain connectivity fingerprints of each voxel in the seed region (here the anterior and posterior PHG; Fig. 8). The tractography‐based parcellation approach yielded four subregions within the right aPHG and two subregions within the right pPHG (Fig. 8A, B). A similar number of subregions were also obtained for the left aPHG and pPHG, demonstrating a symmetric pattern between left and right hemispheres. These PHG clusters exhibited robust cortico-cortical connections (e.g., frontal, occipital, parietal, and temporal) and cortico-subcortical connections (e.g., hippocampus and basal ganglia), demonstrating a caudal-to-rostral trend of fiber projections. Furthermore, the connectivity patterns for each subregion were highly consistent with resting-state functional connectivity (RSFC) patterns obtained from the same neuroimaging data (Fig. 8C, D).

Next, the integrated EEG-fMRI analysis focused on single-trial beta coefficients from the bilateral PHG ROIs and the averaged evoked power at channel E160 during T-maze and No-maze trials. To test the link between the EEG and fMRI data, a set of multimodal LME models were estimated with single-trial BOLD averages for each of the 12 bilateral PHG ROIs as the dependent variables. Thus, one model was run for each ROI in each condition (T-maze vs. No-maze). All *p*-values were corrected for multiple comparisons using the FDR method for each regressor within a model. To test directly our primary hypothesis that single-trial RPT power can predict single-trial PHG BOLD values, we specifically tested the relationship between RPT regressors and BOLD signal from the six PHG ROIs on the right hemisphere (full LME model results for both hemispheres can be found in Supplementary Tables S1–S5).

**Fig. 9.**
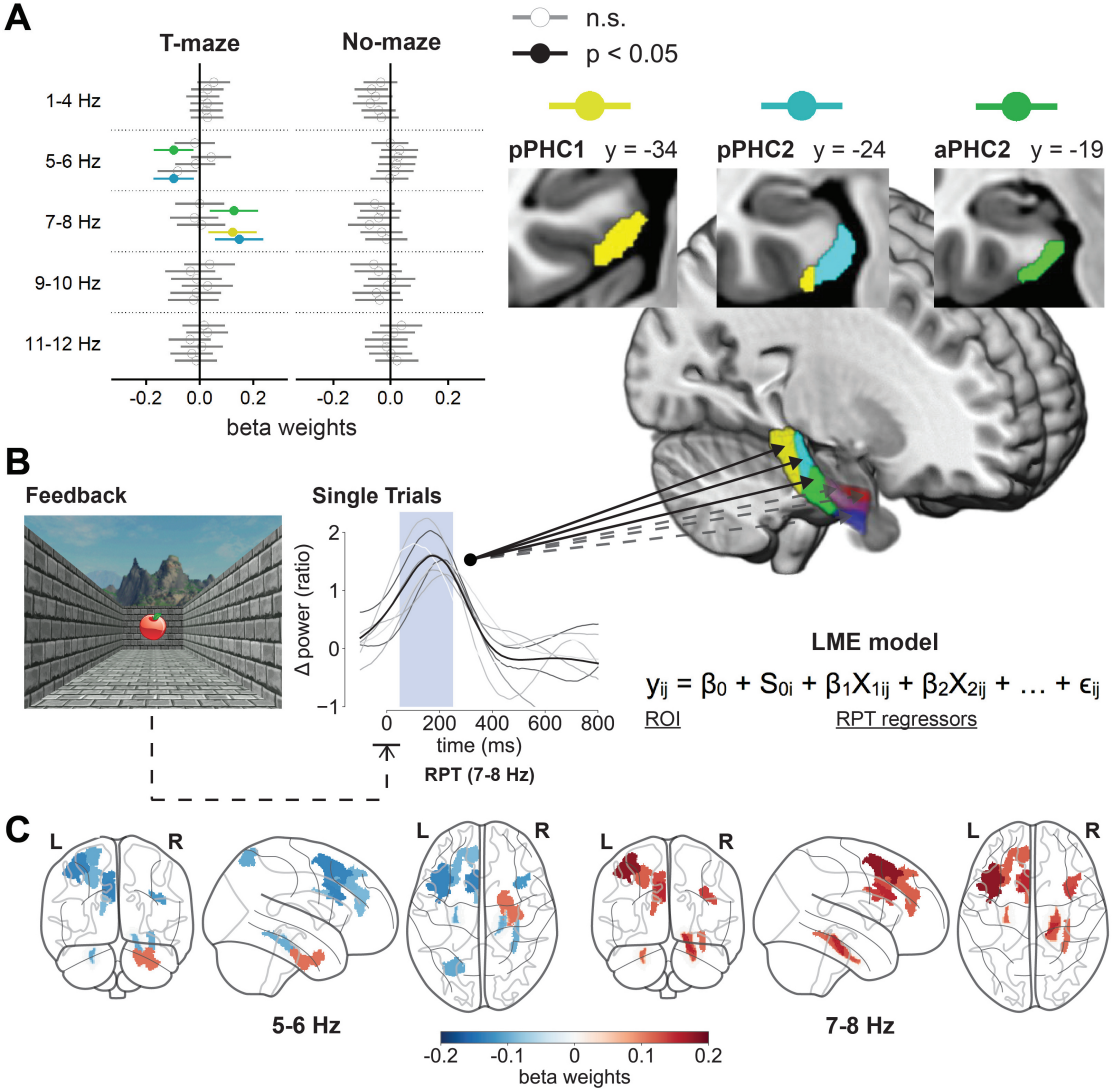
Results from multimodal LME models predicting single-trial BOLD using RPT regressors. (A) Forest plots on the left depict the beta weights for the EEG-based regressors by frequency band and PHG ROI (aPHG2: green, pPHG1: gold, pPHG2: light blue). Left column shows the results for the T-maze condition, right column shows the results for the No-maze condition. The position of circles on the x-axis on each forest plot reflects the size of each weight. Filled out and colored circles are significant beta weights with a *p*-value <0.05 after FDR correction. Gray, empty beta weights are not significant. Lines around dots represent a 95% confidence interval for the respective weight. Sagittal view and coronal slices on the right show the PHG clusters that were significantly predicted by at least one theta frequency band. (B) Bottom panels represent a schematic overview and the equation of the LME models. Line plots depict feedback-locked example trials recorded at E160 (gray, transparent lines) and their average (black line) for subject 8. The mean amplitude from 50 to 250 ms (blue window) was used to build regressors. In each model, y (BOLD value for a given subject i and trial j) was predicted by the overall model intercept β_0_, a random intercept S_0_ for each subject, a fixed regressor β_1–5_ for each EEG frequency band that is weighted by the power for a given trial j and subject i, and a corresponding error term ϵ. Solid arrows denote significant relationships between single-trial EEG regressors and BOLD signals, dashed and transparent arrows denote non-significant relationships. (C) Color-coded PHG beta weights from the LME models run for each Glasser ROI along with the PHG ROIs. Results are separated by weights for the 5–6 Hz regressors (left) and 7–8 Hz regressors (right). All weights are for the T-maze condition.

Single-trial BOLD values in aPHG2 (*t* = 2.81, *p* = 0.03, *b* = 0.129), pPHG1 (*t* = 2.691, *p* = 0.043, *b* = 0.123), and pPHG2 (*t* = 3.207, *p* = 0.008, *b* = 0.148) were significantly predicted by 7–8 Hz evoked power recorded from the right parietooccipital EEG sensor E160 (Fig. 9). The 7–8 Hz regressor received a positive weight for both ROIs, indicating that for a given trial, increases in PHG activation were coupled to increases in 7–8 Hz power. The frequency band below, 5–6 Hz, was also significantly coupled to aPHG2 (*t* = -2.559, *p* = 0.032, *b* = -0.097), and pPHG2 (*t* = -2.522, *p* = 0.024, *b* = -0.097) but with each regressor receiving a negative weight. Hence, a trial-to-trial increase in activation across these PHG ROIs was associated with a decrease in 5–6 Hz power. An overview of all beta weights for the 5–6 and 7–8 Hz theta bands plotted onto their respective ROIs can be found in Supplementary Figure S5. None of the remaining delta and theta bands received significant beta weights. Table 2 summarizes the full results for the right PHG models (left PHG models can be found in Supplementary Table S1). Importantly, among the No-maze condition LME models, none of the aforementioned relationships between single-trial RPT power and PHG BOLD activation were found (see Supplementary Tables S2 and S3), suggesting that the RPT-PHG associations predominantly emerged during spatial (vs. non-spatial) feedback. As a control analysis, we used the same RTP power regressors to predict single-trial BOLD activation in the precuneus (whole brain cluster from Fig. 7A) and the middle superior temporal gyrus (i.e., Heschl’s Gyrus). Neither exhibited significant relationships with RPT power regressors (Supplementary Table S4), demonstrating that the above couplings were specific to the PHG ROIs.

**Table 2.** Fixed effects table of the LME model run on single-trial right PHG ROI BOLD activation (columns) recorded during the T-maze condition.

Right PHG, T-maze condition																		
	aPHG1			aPHG2			aPHG3			aPHG4			pPHG1			pPHG2		
*Predictors*	*Beta*	*t*	*p*	*Beta*	*t*	*p*	*Beta*	*t*	*p*	*Beta*	*t*	*p*	*Beta*	*t*	*p*	*Beta*	*t*	*p*
1–4 Hz	0.054	1.703	0.267	0.030	0.953	0.568	0.013	0.411	0.896	0.028	0.883	0.880	0.026	0.827	0.555	0.030	0.948	0.515
	(-0.008 –0.115)			(-0.031 –0.090)			(-0.048 –0.074)			(-0.034 –0.089)			(-0.035 –0.086)			(-0.032 –0.091)		
5–6 Hz	-0.017	-0.448	0.827	-0.097	-2.559	**0.032**	0.044	1.147	0.896	-0.015	-0.392	0.880	-0.082	-2.163	0.092	-0.097	-2.522	**0.024**
	(-0.093 –0.058)			(-0.171 – -0.023)			(-0.031 –0.119)			(-0.090 –0.060)			(-0.156 – -0.008)			(-0.172 – -0.021)		
7–8 Hz	0.001	0.015	0.988	0.129	2.810	**0.030**	-0.020	-0.435	0.896	0.007	0.151	0.880	0.123	2.691	**0.043**	0.148	3.207	**0.008**
	(-0.090 –0.092)			(0.039 –0.219)			(-0.110 –0.070)			(-0.084 –0.098)			(0.033 –0.213)			(0.058 –0.239)		
9–10 Hz	0.039	0.793	0.827	-0.034	-0.717	0.568	-0.011	-0.235	0.896	0.030	0.621	0.880	-0.013	-0.278	0.781	-0.023	-0.471	0.754
	(-0.057 –0.134)			(-0.129 –0.060)			(-0.106 –0.083)			(-0.065 –0.125)			(-0.108 –0.081)			(-0.118 –0.072)		
11–12 Hz	0.016	0.400	0.827	0.029	0.728	0.568	-0.036	-0.887	0.896	0.009	0.215	0.880	-0.029	-0.736	0.555	-0.013	-0.313	0.754
	(-0.063 –0.096)			(-0.049 –0.108)			(-0.115 –0.043)			(-0.071 –0.088)			(-0.108 –0.049)			(-0.092 –0.067)		

Fixed effect predictors include four EEG regressors (rows) based on evoked delta power (1–4 Hz) and four RPT bands (5–6, 7–8, 9–10, and 11–12 Hz). All listed *p*-values were corrected for multiple comparisons within a model. Significant *p*-values (*p* < 0.05, FDR-corrected) are printed bold next to the corresponding t-value. For each weight, a 95% Confidence Interval (CI) is given below the standardized beta estimate.

Finally, to investigate the specificity of this RPT and PHG coupling, we extended the analysis to the whole brain level by running LME models with the same four EEG regressors predicting single-trial BOLD activation in the T-maze condition for each ROI in the Glasser atlas (Glasser et al., 2016). If RPT power is specifically connected to the PHG and spatial navigation, EEG-based RPT regressors should not predict any nearby temporal or parietal regions unrelated to spatial navigation (e.g., superior temporal gyrus). Figure 9C highlights the significant results of this whole brain analysis for all Glasser ROIs in addition to our PHG ROIs specifically for the two previously significant theta bands, 5–6 and 7–8 Hz (for all regressors, see Supplementary Tables S5 and S6). Almost all signs matched the PHG results, with all but one 5–6 Hz weights receiving a negative sign and all 7–8 Hz weights receiving positive signs. In the right hemisphere, two memory-related structures in the medial temporal lobe laterally to the PHG had significant beta weights: one laterally adjacent to our pPHG definition (Glasser label: “Parahippocampal Area 3”) for both 5–6 Hz (*t* = -2.453, *p* = 0.045, *b* = -0.097) and 7–8 Hz (*t* = 2.435, *p* = 0.045, *b* = 0.112) and one in the perirhinal cortex (“Ectorhinal-Perhinal Cortex”) only for 5–6 Hz (*t* = 2.898, *p* = 0.023, *b* = 0.109). Note that the Glasser atlas employs an anatomical delineation of the PHG that laterally extends into and past the collateral sulcus and thus includes regions that we did not include in our definition (e.g., “Parahippocampal Area 3”). No other significant relationships were identified in the temporal and parietal lobe for the right hemisphere. Furthermore, six left hemispheric and two right prefrontal regions, including areas relevant to feedback processing such as the left anterior cingulate cortex and the dorsolateral prefrontal cortex, displayed significant relationships with 5–6 and 7–8 Hz (for full results, see Supplementary Tables S5 and S6).

### 3.3 Discussion—Experiment 2

We recently demonstrated that the presentation of feedback-related stimuli in a virtual environment induces a burst of EEG oscillations in the theta frequency range over right posterior areas of the human scalp (RPT), and that the power and phase angle of RPT were significantly larger when participants receive feedback following right turns in the maze compared with left turns in the maze. Even though source localization procedures have indicated that the RPT is produced in or near the PHG (Baker & Holroyd, 2013), and, even though the tasks previously employed to produce the RPT effect have generated a strong hemodynamic response in the right PHG (Baker et al., 2015), the origin of RPT still remains uncertain (see Supplementary Table S7 for an overview of previous studies). This may be due to the inherent spatial limitations of EEG recordings and the lack of time–frequency information in fMRI data. These limitations are formally insoluble but can be ameliorated by the application of the “converging methods” approach in which multiple source analysis techniques are utilized to compensate for their respective weaknesses (Luck, 1999, 2005, p. 296). We hypothesized that converging evidence using simultaneous EEG-fMRI data recorded during the virtual T-maze task would provide strong evidence that RPT is in fact generated in the PHG.

First, to test whether RPT depends on spatial processing rather than other aspects of task performance, we had participants engage in two tasks: the standard T-maze task and a No-maze task. The No-maze task was formally identical to the T-maze but was devoid of the spatial context. As predicted, the RPT effect (latency difference between left and right alleys) occurred only when participants processed the feedback in the maze environment, ruling out other potential sources of the effect such as dependence on right versus left button presses. The power of RPT was equivalent across the two tasks, suggesting that the underlying neural system was fully engaged even in the No-maze task. Thus, despite the small size of the latency difference (approximately 50 ms), these results replicate our previous work (Baker & Holroyd, 2009, 2013). Notably, several studies have shown that neighboring regions of the PHG, particularly the fusiform gyrus (Iidaka et al., 2006; Rossion et al., 2003), play an important role in object recognition. We proposed that RPT was generated by this network for object processing in the ventral-medial temporal cortex, and that the associated right-hemispheric dominance and latency effect occurred when this processing stream was modulated by spatial context.

Second, consistent with our predictions for the fMRI results, we found that the right PHG was more strongly activated by feedback stimuli encountered in the same virtual T-maze that we utilized in our previous EEG studies, when compared with feedback stimuli presented with no spatial context (i.e., No-maze task). Further, statistically significant activations were also observed in the left PHG, bilaterally in the middle temporal-occipital cortex, and in the right precuneus (Fig. 7; Table 1), regions commonly activated during virtual navigation, and consistent with our previous report (Baker et al., 2015). In particular, precuneus activity in fMRI studies has been associated with spatial navigation (Lithfous et al., 2017; Ohnishi et al., 2006), self-referential representations during episodic memory (Hebscher et al., 2018), mental imagery (Cavanna & Trimble, 2006), and it has been reported as a part of the default-mode network (Utevsky et al., 2014). It is often associated with allocentric spatial memory and memory retrieval (Frings et al., 2006; Wallentin et al., 2008; H. Zhang & Ekstrom, 2013), but also with the updating of egocentric spatial information that pertains to an individual’s position relative to motivationally relevant objects (Dordevic et al., 2022; Galati et al., 2000).

Third, we predicted that in the T-maze, the right PHG would generate a stronger hemodynamic response to feedback stimuli encountered in the right alley compared with the left alley of the T-maze. Our results confirmed this prediction. Consistent with our previous fMRI study, the current analysis revealed two distinct regions within the right PHG: in the T-maze versus No-maze contrast, a large significant cluster was observed in the pPHG, whereas in the right alley versus left alley contrast, a large pPHG and a small aPHG cluster of activation were noted. In contrast to the viewpoint that the PHG is a single coterminous region that performs a unitary spatial function, our findings support the idea that the PHG is composed of multiple functional components along its posterior-anterior axis. For example, the Parahippocampal Place Area (a region reported to occupy the posterior portion of the PHG and extending into the lingual gyrus) responds preferentially to pictures of places (Epstein et al., 1999, 2003), the posterior and middle regions of the PHG are involved in the encoding and retrieval of information relevant to navigation (Huntgeburth et al., 2017; Janzen & Van Turennout, 2004; Sulpizio et al., 2013), and the entorhinal cortex (occupying the anterior portion of the PHG) has been shown to play a more general role in memory (Mankin et al., 2021; Owen et al., 1996). Moreover, evidence from functional connectivity studies supports the idea that aPHG and pPHG exhibit distinct connectivity patterns, with the pPHG exhibiting a stronger relationship with visual regions in the occipital lobe and the aPHG being more strongly coupled with parietal lobe regions implicated in spatial processing (Baldassano et al., 2013; D. M. Watson & Andrews, 2024; Zhuo et al., 2016). Consistent with this view, our analysis of an HCP dataset showed that subregions within the PHG were diversly connected to unimodal and polymodal association areas as well as frontal regions. In particular, this analysis revealed connections that included the medial frontal cortex and anterior midcingulate via the cingulum bundle, as well as the medial occipital and temporal cortices and the precuneus via the posterior corona radiata.

The above detailed analyses served largely to replicate our previous EEG and fMRI findings. In addition, the main purpose of the study was to test whether trial-by-trial variation in RPT power was coupled to trial-by-trial variation in PHG BOLD activation, which would implicate the right PHG in producing RPT. Consistent with our prediction, we observed that trial-by-trial variation in theta power in the 7–8 Hz range predicted trial-by-trial fMRI activation in two clusters in the right pPHG and one cluster in the right aPHG. This result suggests that with increased BOLD signals in the PHG, subjects exhibited enhanced power in RPT (7–8 Hz), supporting the hypothesis that the identified PHG clusters are the plausible generators of RPT recorded at the scalp. Importantly, there were no significant relationships between RPT and PHG activation during the No-maze condition in any of the LME models. This highlights that the theta-linked PHG engagement may be specific to spatial navigation. Furthermore, in our control and whole brain analyses, most significant couplings between BOLD signal and RPT in the right temporal and parietal cortical areas were exclusive to the medial temporal lobe, demonstrating that the significant RPT and BOLD couplings were specific to our hypothesized regions. Significant relationships between RPT and primarily left prefrontal regions (e.g., anterior cingulate cortex, dorsolateral prefrontal cortex) likely stemmed from these areas being co-activated with the PHG due to their involvement in feedback processing (Baker, Lesperance, et al., 2017; Becker et al., 2014; Liu et al., 2011; Marco-Pallarés et al., 2007; Sescousse et al., 2013). However, given their distance to the electrode site where RPT is recorded (E160), these cortical areas are unlikely sources of RPT. Instead, they could be part of the spatial navigation network activated as a result of phase resetting in the PHG propagating spatial feedback information. This idea is supported by our findings of strong structural and functional connectivity between our PHG ROIs and prefrontal cortices.

An unexpected finding in this study was that RPT between 5 and 6 Hz was significantly negatively coupled to almost all the ROIs that were positively associated with 7–8 Hz. This suggests that the unique variances of 5–6 and 7–8 Hz were inversely related to PHG BOLD, where 7–8 Hz increased with heightened BOLD on a given trial while 5–6 Hz decreased. A possible explanation is that phase resetting could result in the synchronization of oscillations in the preferred frequency range of 7–8 Hz for optimal encoding of reward locations, while the immediately adjacent frequencies such as 5–6 Hz on a trial-by-trial level are suppressed. The need for the tuning of encoding-related theta frequencies has been demonstrated by Quirk et al. (2021) who created memory encoding deficits in mice searching a maze for navigational cues by shifting the frequency of endogenous theta rhythm of hippocampal and entorhinal grid cells. Pushing the endogenous theta rhythms of grid cells (6–9 Hz) above their preferred range decreased the animals’ ability to encode reward information. Such frequency tuning toward an optimal range depending on task demands has also been reported for prefrontal EEG theta oscillations in humans (Senoussi et al., 2022). In addition, the 5–6 Hz RPT power as observed in our experiments could stem at least partially from entorhinal and perirhinal cortex activity, which in our whole brain analysis was the only ROI with a positive coupling to 5–6 Hz, while the PHG’s rhythm gravitated toward 7–8 Hz at the time of the phase reset. To note, single-trial 5–6 and 7–8 Hz RPT power recorded over the left hemisphere (E107) significantly predicted left anterior PHG activation with the same signs as for the right hemisphere. Given that RPT is marked by a bilateral asymmetric increase in theta power and a bilateral PHG activation in the fMRI whole brain contrast, couplings in the same frequency ranges with the same signs for both hemispheres are in line with our hypothesis of the PHG underlying RPT. This finding indicates that more anterior PHG regions with a more general role in memory and no sensitivity to direction produce theta activity in the left hemisphere.

## 4 General Discussion

A large body of evidence from rodent studies indicates that the PHG updates and stores contextual information related to spatial navigation according to the phase of local electrophysiological oscillations in the theta frequency range. In the present study, we provide converging evidence from EEG, MEG, and simultaneous EEG-fMRI data supporting the proposal that RPT elicited by feedback received in the virtual T-maze reflects phase resetting of the ongoing theta rhythm in the right PHG, and that the RPT phase shift between feedback presented in left and right alleys results from differences in the timing of the theta phase reset. Experiment 1 replicated the RPT effect and observed an analogous MEG component in the same group of individuals performing the virtual T-maze task. Consistent with previous work, feedback presented in the right alleys elicited an earlier and larger RPT peak, a larger ITC peak in the RPT range of 7–10 Hz, and a higher phase alignment in the MEG than left alleys. Importantly, since the MEG data yielded the same results as the EEG data and previous EEG studies, our EEG findings are likely not a result of volume conduction since MEG is less susceptible to this distortion (van den Broek et al., 1998). Moreover, with the MEG’s higher spectral resolution, we can now more confidently state that the RPT effect is in the suspected 7–10 Hz range. Crucially, in Experiment 2, this range exhibited the only positive single-trial coupling to the right PHG, strengthening the proposal that the RPT effect related to partial phase resetting is linked to the PHG.

These findings are consistent with decades of animal and computational research showing that theta phase resetting occurs across the medial temporal lobe in response to motivationally relevant spatial information (Givens, 1996; Hasselmo et al., 2009), which supports the encoding of goal-related spatial information (Burgess, 2008; Burgess et al., 2007; McCartney et al., 2004; Williams & Givens, 2003). Our findings also match those of studies examining human intracranial recordings, which report the existence of PHG cells producing navigational theta oscillations sensitive to landmarks (Ekstrom et al., 2005). In addition, our results provide further evidence for phase resetting occurring across temporal and frontal cortical areas during memory encoding and retrieval (Rizzuto et al., 2006), and for theta power being modulated by the relevance of spatial information to goal attainment (Stangl et al., 2021). Phase resetting has been proposed to reflect a crucial neural mechanism for synchronizing oscillations by aligning their phase to relevant reference points (Canavier, 2015). For example, according to the oscillatory interference model (Burgess, 2008; Burgess et al., 2007), neural representations of spatial locations are reflected in the relative phases of theta local field potentials in the hippocampus and PHG. In this model, phase resetting serves as a mechanism for establishing place cells, changing their activity when new information is discovered (i.e., rewards), and correcting error accumulation between the different oscillators. Accordingly, human phase resets in response to spatial information, as indexed by RPT, could serve to create spatial representations around motivationally relevant locations that contain rewards and coordinate this information across larger networks. This interpretation is consistent with a common account describing phase resetting and cross-frequency coupling as tools for balancing local population activity across regions of a network (Voytek & Knight, 2015).

In previous studies that employed the virtual T-maze task, as well as in our present examination, analyses of EEG, MEG, and fMRI data have been pointing consistently to a difference in processing of feedback stimuli in right alleys as opposed to left alleys. This was reported to occur only when navigating in the T-maze condition (Baker et al., 2015; Baker & Holroyd, 2009, 2013). Taken together, these results represent a rightward bias where the PHG responds more sensitively and earlier to information discovered following rightward trajectories. While the present results and existing literature point toward bilateral spatial processing (Ohnishi et al., 2006; Weniger et al., 2010), they are also consistent with the long-standing observation of right hemisphere dominance (Jacobs et al., 2010; Maguire, Burgess, et al., 1998; Toussaint et al., 2008; Weniger & Irle, 2006). With the current data, we cannot draw any conclusions and can only speculate as to where this bias for rightward trajectories stems from. We previously proposed that during this 2D virtual T-maze task, the two goal locations in the left and right alleys are represented by the same place field, since subjects remain stationary during the task and process the T-maze as a single location (M.-H. Lin et al., 2022). As a result, the two goal locations could be represented by different phase positions along the theta cycle of a single place field, which in turn resets the phase of the PHG theta oscillation at different timings. Future research will have to build on these speculations to determine their accuracy.

## 5 Limitations

It is important to note as a limitation of this study that while most of the replications were successful, we only replicated the difference in phase alignment between left and right alleys in the EEG data of Experiment 1 with the ITC analysis but not the RVL analysis. This may be due to the far smaller sample size (n = 11) and decreased power of this experiment compared with our previous study (n = 91; Baker & Holroyd, 2013). It is likely that this phase alignment difference in the EEG would have reached significance in a larger sample, given that the spatial left and right alley difference was successfully replicated in both the EEG and MEG studies when analyzing the ITC, RPT power, and latency.

Similarly, Experiment 2 mostly replicated previous findings successfully. However, in the EEG data, there was no significant RPT power difference observed between left and right alleys in the T-maze condition. Furthermore, the fMRI voxel clusters in the aPHG exhibited only weak activation for the left versus right alley contrast. For the latter, smaller clusters with relatively small activation compared with the pPHG were in line with our previous findings, further emphasizing the dominant involvement of the pPHG in processing feedback in the virtual T-maze task. Regarding the EEG data, the lack of power difference could be attributed to the heavy artifact correction procedures due to the conditions when recording EEG inside the MRI scanner. Yet, given that the spatial distribution of RPT, its timing, and latency difference between left and right alleys specific to the T-maze condition were consistent with results observed in previous studies, we consider this a successful replication. Regarding the single-trial analysis and multimodal PHG parcellation, it is notable that among the PHG ROIs significantly predicted by RPT power, two were in the pPHG and one was in the aPHG (i.e., aPHG2), exhibiting the same trends as the pPHG. Given this result and the overlap in connectivity between aPHG2 and posterior PHG ROIs, it is possible that aPHG2 and pPHG1 are part of the same region that were identified as unique due to individual differences between subjects and spatial smoothing.

Finally, while the current results support our hypothesis of RPT being related to theta phase resetting in the PHG, future research will have to replicate our combined EEG-fMRI findings while counterbalancing low-level visual features such as textures of the Maze/No-maze task (Ghodrati et al., 2016; Groen et al., 2012; Sutterer et al., 2021), as we have done in the past (Baker & Holroyd, 2009). Furthermore, future studies will need to explore the relationship between RPT and memory encoding as well as how information is processed in the hypothesized regions connected to the PHG in a larger spatial navigation network. Previously, we found that the timing of the ERP peak related to RPT and PHG BOLD activation during the virtual T-maze task were correlated to how well subjects recalled the shape of the maze they had navigated during the recordings (Baker et al., 2015; Baker & Holroyd, 2013). Together, these findings suggest that RPT is specific to moving in spatial environment and that RPT reflects interindividual variability in how subjects process environmental cues with respect to movement through an egocentric frame of reference. However, empirical tests of the relationship between RPT and memory encoding are still needed.

## 6 Conclusion

The present study adds support for the proposal that feedback-related RPT power is a scalp level signature for the human PHG theta phase reset in service of goal-directed spatial navigation. Specifically, these results strengthen the idea of the PHG as the source of the human RPT effect, which has substantial implications for the clinical research and diagnostics of memory-related disorders. For example, with declines in spatial navigation abilities gaining attention as a potential screening tool for the detection of early-onset Alzheimer’s disease (Allison et al., 2019; Coughlan et al., 2018), the associated biomarkers have also garnered interest in the attempt to achieve higher diagnostic precision and sensitivity (Brueggen et al., 2017; de Toledo-Morrell et al., 2000). The present theta PHG signature has the potential to serve as a scalp-level signature of the integrity of functions like memory encoding linked to phase resetting. Future work could make use of this in guiding the diagnostic process or for non-invasive neurostimulation interventions in memory-related disorders, such as Alzheimer’s disease.

## Supplementary Material

Supplementary Material

## Data Availability

Analysis scripts, documentations, examples for preprocessing and higher level statistics, and the 12 bilateral PHG ROIs as a binary nifti files can be found in the github repository for this study: https://github.com/BakerlabRutgers/phg_phase_resetting. All further code and data are available upon reasonable request.
