# Supplementary material for: Right posterior theta reflects human parahippocampal phase resetting by salient cues during goal-directed navigation
Source: Imaging Neurosci (Camb). 2025 Sep 8;3:IMAG.a.105. doi: 10.1162/IMAG.a.105 (PMC12418113; doi:10.1162/IMAG.a.105)
Supplement: Supplementary Material [file IMAG.a.105_supp.pdf]

Supplementary Material

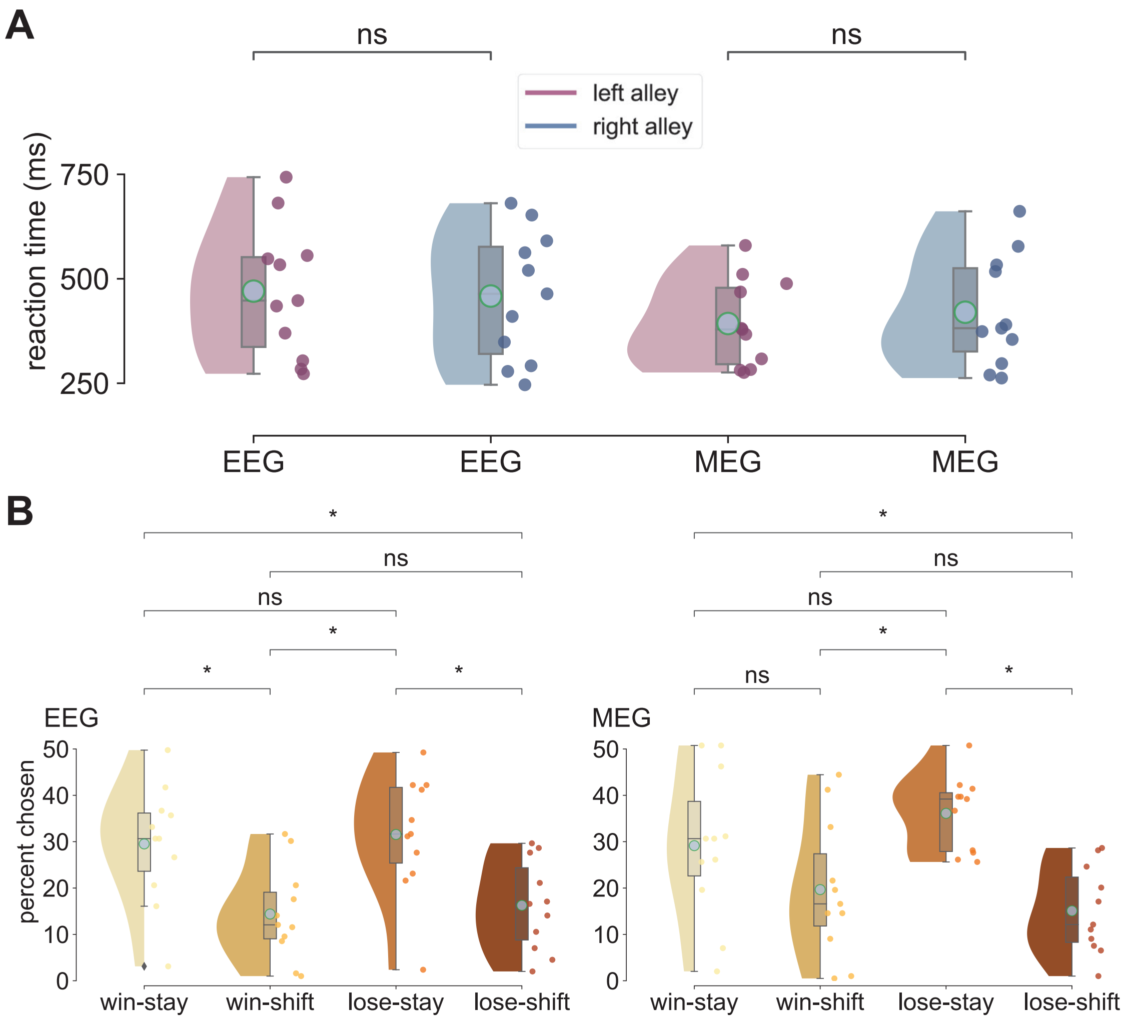

Supplementary Figure S1. Experiment 1: Behavioral data analysis. A: Raincloud plots show RT density distributions with boxplots and individual subject averages in milliseconds (ms) plotted separately for each Feedback Alley Type (left in pink, right in blue) and modality. Cyan dots mark the mean value of each distribution. As has been reported before, right and left alleys in both EEG and MEG were behaviorally equivalent. In the EEG session, there was no significant difference between alleys in regard to how frequent either alley was chosen (W = 31, p = 0.859). In regard to reaction times, right alleys (M = 459 ms, SD = 147 ms) were chosen at approximately the same speed as left alleys (M = 470 ms, SD = 151 ms), W = 17, p = 0.154. During the MEG session, subjects also did not choose either alleys more frequently than the other (W = 19, p = 0.213) and responded with approximately the same reaction times for right (M = 420 ms, SD = 127 ms) as for left alleys (M = 392 ms, SD = 100 ms), W = 13, p = 0.075. **B**: Percentage of response strategies relative to total choices. Same as in A, cyan dots are the mean of each distribution and dots colored like the density plots are each individual subject's mean value. In both the EEG and MEG session, the two most prevalent patterns were win-stay and lose-stay, each accounting for roughly 30% of total responses. In the EEG session,

there was a significant main effect for the factor response strategy, $Q = 16.1$, $p = 0.001$. Consistent with previous experiments, post-hoc tests showed that win-stay when compared to win-shift was significantly more frequent ($W = 6$, $p = 0.016$). However, unlike in previous experiments, lose-shift was a significantly more common response strategy than lose-stay ($W = 5$, $p = 0.022$), indicating a tendency for sticking to the same alley regardless of feedback. A similar pattern of results was observed for the MEG session, with a significant main effect for response strategy ($Q = 10.96$, $p = 0.012$) and win-shift and lose-stay as the more common response strategies. Here, win-stay and win-shift were not significantly different ($W = 19$, $p = 0.213$), but lose-stay was significantly more frequent than lose-shift ($W = 3$, $p = 0.008$). *ns: not significant, *p < 0.05*

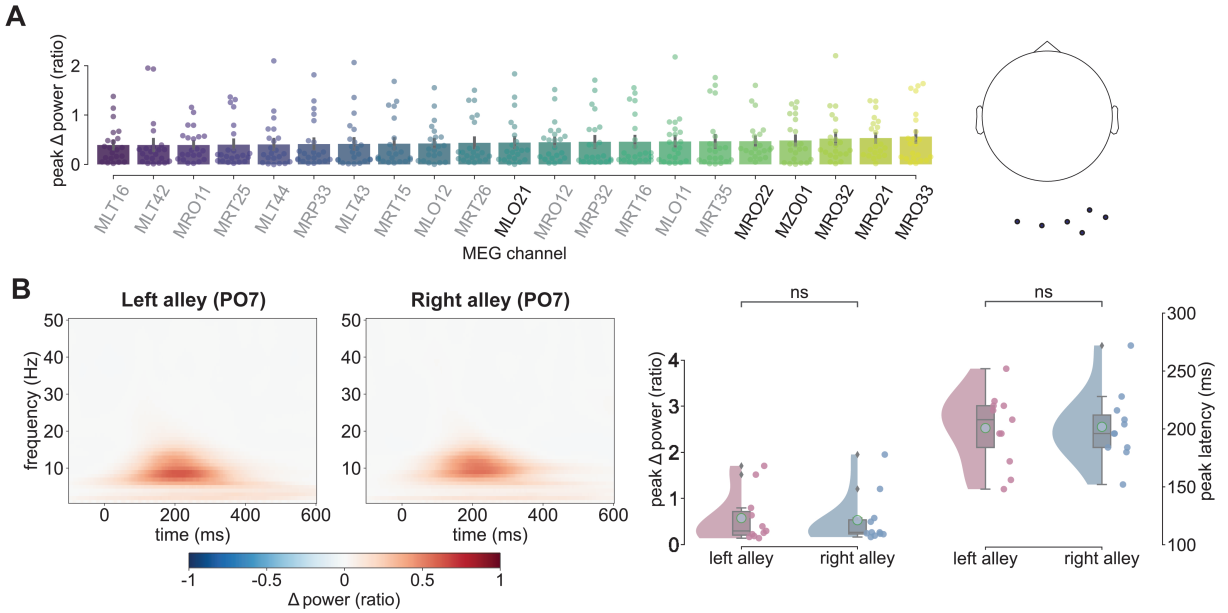

Supplementary Figure S2. MEG peak RPT channel statistics and EEG left hemisphere RPT results. A: Bar plots highlight the 20 MEG channels with the highest peak evoked RPT power post-feedback averaged across conditions and subjects. Since this experiment is the first application of the virtual T-maze task while recording MEG, channels for statistical analysis were identified based on an exploratory approach. For each subject the channel carrying the peak RPT was extracted. Bar plots with black labels mark channels that were picked at least for one subject. The 2D topoplot on the right shows the locations of these channels identified as peak channels. **B**: Spectrograms with evoked power change relative to baseline (expressed as ratio) from -100 ms to 600 ms post-feedback separated by alleys (left alleys vs. right alleys) at EEG channel PO7. Raincloud plots show the distributions of theta power peaks and theta power peak latencies by alley (left in pink, right in blue) for PO7. Cyan dots mark the mean value of each distribution. Red and blue dots represent individual subject values. **ns: not significant*

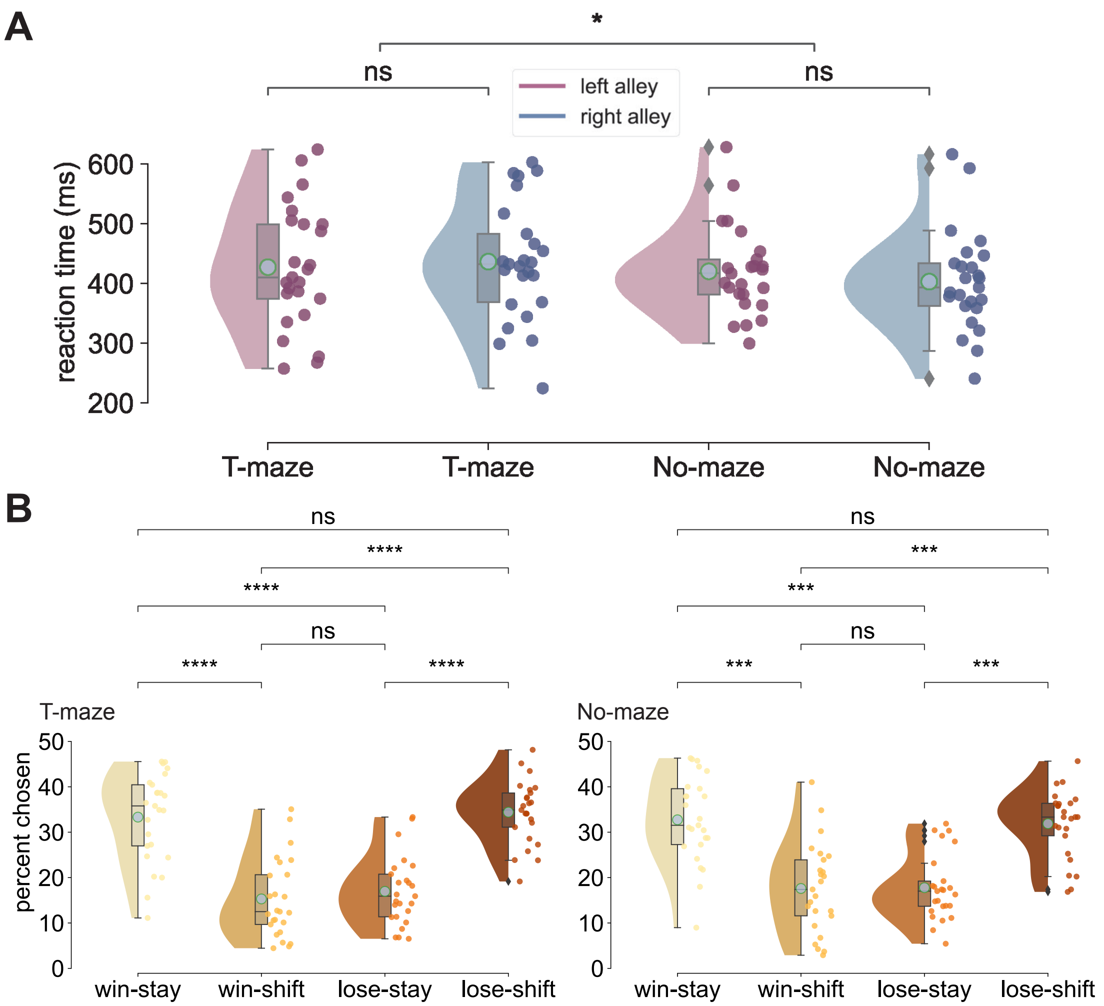

Supplementary Figure S3. **Experiment 2: Behavioral data analysis. A**: Raincloud plots depicting the distribution of individual subject averages in milliseconds (ms) separated by alley (left in pink, right in blue) and condition (T-maze vs. No-maze) with cyan dots indicating the mean value of each distribution. A two-way repeated measures ANOVA run on the sum of left and right choices yielded neither a significant main effect of Feedback Alley Type ($F(1, 24) = 0.478$, $p = 0.495$, $\eta_p^2 = 0.019$) nor an interaction between Feedback Alley Type and Task Type ($F(1, 24) = 0.042$, $p = 0.837$, $\eta_p^2 = 0.001$), indicating that right and left alleys were chosen an equally in both the T-maze and No-maze condition. Similarly, there was no significant main effect of Feedback Alley Type when calculating the same ANOVA for the dependent variable reaction times, $F(1, 24) = 0.453$, $p = 0.507$, $\eta_p^2 = 0.018$. Thus, left and right alleys were equivalent in reaction times. However, in the T-maze condition (M = 432 ms, SD = 95 ms) subjects responded significantly faster than in the No-maze condition (M = 412 ms, SD = 75 ms), as revealed by a significant main effect of the factor Task Type, $F(1, 24) = 4.37$, $p = 0.047$, $\eta_p^2 = 0.154$. **B**: Percentage of response strategies relative to total choices. Same as in A, cyan dots are the mean of each distribution and dots colored like the density plots are each individual subject's mean value. Consistent with previous studies, win-stay (M = 33%, SD = 10%) and lose-shift (M = 33%, SD = 7%) emerged as the most common response

strategies. This was confirmed by a significant main effect of the factor response strategy ($F(1, 24) = 27.24$, $p = 1.04 \times 10^{-7}$, $\eta_p^2 = 0.531$) in a two-way repeated measures ANOVA, testing the effects of the factors response strategy (win-stay, win-shift, lose-stay, lose-shift) and Task Type. Post-hoc tests (Bonferroni-Holm corrected) revealed that specifically win-stay was more frequent than win-shift ($t(24) = 4.86$, $p = 8.78 \times 10^{-5}$) and lose-stay ($t(24) = 6.28$, $p = 5.18 \times 10^{-6}$), and that lose-shift was more frequent than win-shift ($t(24) = 6.37$, $p = 5.18 \times 10^{-6}$) and lose-stay ($t(24) = 5.85$, $p = 9.87 \times 10^{-6}$). *ns: not significant, ***p < 0.0001, ****p < 0.00001*

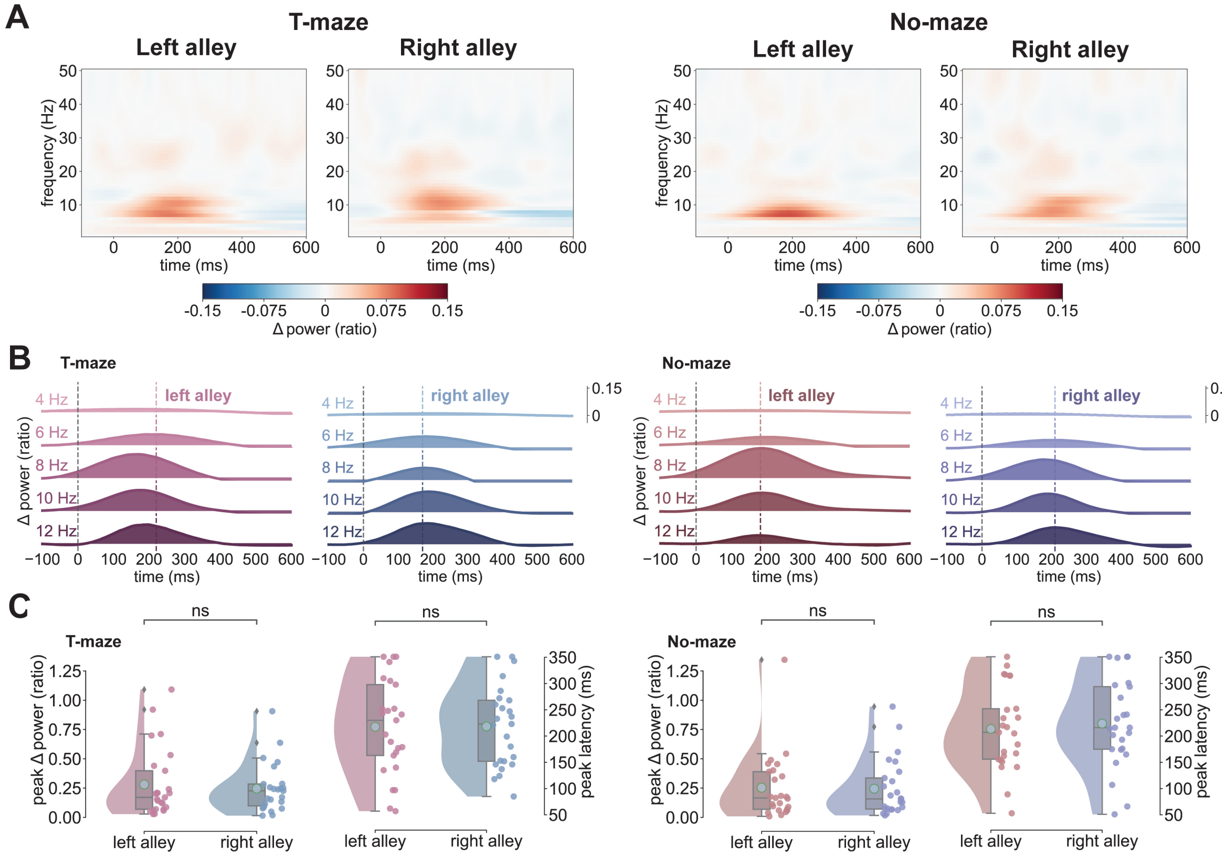

Supplementary Figure S4. **Left hemisphere RPT results. A**: Spectrograms depicting the evoked power from -100 ms to 600 ms post-feedback at E107 (left hemisphere) separately for Task Type (T-maze: left panels, No-maze: right panels) and alley (left alleys vs. right alleys). Unlike on the right hemisphere, the two-way repeated measures ANOVA run for RPT peak latency with the factors Task (T-maze vs. No-maze) and Feedback Alley Type (left vs. right alley) neither yielded a significant interaction effect ($F(1, 24) = 0.113$, $p = 0.739$, $\eta_p^2 = 0.005$) nor any significant main effects. Left alleys were followed by RPT peak latencies (M = 218 ms, SD = 89 ms) as right alleys (M = 218 ms, SD = 75 ms) in the T-maze and in the No-maze (left: M = 214 ms, SD = 77 ms; right: M = 224 ms, SD = 82 ms). The same was observed for the ANOVA run on RPT peak amplitudes. **B**: Ridge plots show the same results as in A divided into the delta band (1-4 Hz) and theta bands (5-6 Hz, 7-8 Hz, 9-10 Hz, and 11-12 Hz), with the average RPT peak timing for each condition marked by a dashed colored line. Results for the T-maze condition are on the left side and for the No-maze condition on the right side. **C**: Raincloud plots depict the RPT power

peaks and peak latencies for the T-maze condition on the left and the No-maze condition on the right, with cyan dots showing the mean value of each distribution. Alley is shown on the x-axis and colored dots represent individual subject values. ns: not significant

PHG segmentation analysis: Diffusion-weighted and resting-state functional MRI

Participants and data acquisition We investigated the anatomical and functional organization of the human PHG using diffusion and functional MRI data from the HCP. We performed a probabilistic tractography analysis on diffusion-weighted data from the HCP to form a parcellation of the anterior PHG (aPHG) and the posterior PHG (pPHG) based on whole brain connectivity that is conform with anatomical delineations of the PHG and matches RSFC profiles of the PHG. Data were obtained from the 500-subject release of the HCP (HCP RRID:SCR_008749) database from March 2015. The multimodal data used here include structural MRI, diffusion-weighted MRI, and resting-state MRI. The scanning procedures are described in detail in Van Essen et al. (2012) and are available online (https://www.humanconnectome.org). In total, 430 subjects' data were preprocessed and passed quality control. All data processing was done using our own analyses without the preprocessing by the HCP.

Seed regions The seeds of the anterior and posterior PHG regions were defined manually by reference to the morphology of the sulci of the collateral sulcal complex as described by Huntgeburth and Petrides (2012) on an average normalized (ICBM152) anatomical scan using the DISPLAY software package (MacDonald, 1996). In brief, the posterior boundary of the PHG, and thus the parahippocampal cortex, is based on the junction between the collateral sulcus proper and the PHG extension of the collateral sulcus. The point where the PHG extension and the collateral sulcus proper merge provides the point of origin of the occipital extension of the collateral sulcus which continues into the lingual gyrus, thereby forming the posterior limit of the PHG. In regards to the anterior boundary of the PHG, the rhinal sulcus forms the lateral limit of the entorhinal cortex that is located on the anterior portion of the PHG, with the caudal end of the rhinal sulcus marking the posterior border of the entorhinal cortex. The PHG extends posterior to the entorhinal cortex and, therefore, the rostral origin of the anterior segment of the collateral sulcus proper provides an anatomical marker of the anterior limit of the PHG. Furthermore, the collateral sulcus proper forms the lateral boundary of the

PHG, delimiting it from the adjacent fusiform gyrus. When the collateral sulcus proper can be divided into two sulcal segments, the transition between the anterior and posterior segments occurs approximately at a y-coordinate of -33 in each hemisphere. The two segments of the collateral sulcus proper thus form anatomical landmarks that may be used to differentiate between the aPHG versus the pPHG: the aPHG lying medially along the anterior segment of the collateral sulcus proper, and the pPHG along the posterior segment of the collateral sulcus proper, rostral to the lingual gyrus. Once defined, the aPHG and pPHG masks were linearly registered to native diffusion space.

Diffusion MRI Diffusion data were collected with 1.25 mm isotropic spatial resolution and three diffusion weightings using HCP dMRI protocol (Sotiropoulos et al., 2013) and downloaded using the HCP Diffusion pipeline (Glasser et al., 2013). The probability distributions of fiber orientation were estimated by using FSL's (RRID:SCR_002823) multi-shell spherical deconvolution toolbox (bedpostx), where each voxel contains at most three fiber directions and the diffusion coefficients were modelled using a Gamma distribution (Jbabdi et al., 2012). A T1-weighted image downsampled to the resolution of the diffusion data was employed for the nonlinear registration of the anterior and posterior seed from MNI standard space to native structural volume space using FSL’s package FNIRT. The parcellation of aPHG and pPHG was carried out on 40 randomly selected subjects (20 female, 20 male) in order to limit computation time and data storage. To test the robustness of parcellation, we randomly divided the 40-subjects dataset in half and applied the parcellation procedure (as explained below) independently to each group. The first 20 subjects were used as the test group to reveal the underlying organizational pattern of the PHG and a second group of 20 subjects was subsequently used as the replication group to test the stability of our parcellation maps. All diffusion data (n = 430) were then used during the tractography analysis to map the connectivity profiles of each of the PHG subdivisions identified by the parcellation (please see Zhang et al. (2017) for full details).

Connectivity-based parcellation of PHG A data-driven connectivity-based brain parcellation procedure was used as described in Zhang et al. (2017) (see also Fan et al., (2014, 2016)). First, probabilistic tractography was applied by sampling 5000 streamlines at each voxel within the aPHG and pPHG seed mask. A target mask was constructed for

each subject that included all brain voxels (white or gray matter) connecting to the seed region. The whole brain connectivity profile for each aPHG and pPHG voxel was then saved as a connectivity map and used to generate a connectivity matrix with each row representing the whole brain connectivity profile of one seed voxel. Next, a correlation matrix was calculated as a measure of similarity between the connectivity profiles of each voxel pair with the aPHG and pPHG (Johansen-Berg et al., 2004). Spectral clustering was applied to the similarity matrix to identify clusters with distinct connectivity profiles (Liu et al., 2013; Shi & Malik, 2000). We applied this procedure separately for each subject and each hemisphere to generate a series of parcellation maps for all individuals at different resolutions (i.e. numbers of regions/parcels) and chose cluster numbers ranging from 2 to 6 in each hemisphere, subsequently using the most stable and consistent parcellation map. The optimum parcellation solution (i.e. number of parcels) was then determined by evaluating the reproducibility of parcellation maps through a split-half procedure. More specifically, we randomly split the entire group 100 times into two non-overlapping subgroups and generated the group parcellation maps for each subgroup separately. The consistency between each pair of parcellation maps was evaluated by using the normalized mutual information (P. Zhang, 2015). The average indices among 100 samples were calculated to represent the stability of each parcellation. The suitable cluster number was then determined by searching for the local peaks in the stability curve.

Connectivity profile of each PHG subdivision Based on the obtained parcellation map of the aPHG and pPHG, we mapped the anatomical connectivity profiles of each subdivision by performing probabilistic tractography with 10,000 streamlines from each PHG subdivision. The resulting connectivity maps were first normalized by the size of the seed region and total number of streamlines (i.e. 10,000) in order to generate the relative tracing strength from the seed to the rest of the brain. A threshold of 0.001 (i.e. 10 out of 10,000) was then used to remove noise effects of fiber tracking. The resulting individual tractograms were combined to generate a population map of the major fiber projections for each aPHG and pPHG subdivision. Another probabilistic threshold of 50% was applied to the population fiber-tract maps (i.e. at least half of subjects showing each retained fiber tract). This resulted in a group averaged tractogram for each subdivision of aPHG and pPHG. Finally, a maximum probability map (MPM) of fiber tracts, which represented

distinct components of fiber projections for each subdivision, was also generated based on the population fiber-tract maps. Specifically, a connectome mask was first generated for each subdivision by binarizing its group tractography map with connectivity probability at 0.001. Each voxel within the combined connectome mask was then classified according to the PHG subdivisions with which it had the highest connectivity. This calculation of a MPM on probabilistic tractography has been widely used in subdividing brain structures, including the thalamus (Behrens et al., 2003), amygdala (Saygin et al., 2011), striatum (Cohen et al., 2009), and substantia Nigra (Y. Zhang et al., 2017). Here, we use this method to generate the organizational topography of fiber projections among anterior and posterior subdivisions.

Functional connectivity patterns of the aPHG and pPHG subregions The data used here was the 430 participant subset from the "500 Subjects" HCP release. Preprocessing consisted of standard resting-state functional connectivity preprocessing. Resting-state data collection details for this data set can be found elsewhere. Spatial normalization to a standard template, motion correction, and intensity normalization were already implemented as part of the HCP in a minimally processed version of the data set described elsewhere. With the volume version of the minimally preprocessed data, we used AFNI58 to additionally remove nuisance time series (motion, ventricle and white matter signals, along with their derivatives) using linear regression, to remove the linear trend for each run and spatially smooth the data. The data were smoothed using a non-Gaussian filter (nearest neighbor averaging) at 4 mm. For the main analyses, we used the aPHG and pPHG subregions derived from the connectivity-based parcellation of PHG for the functional connectivity seed regions. Additional regions included two reinforcement-related frontal targets (anterior cingulate cortex and ventromedial prefrontal cortex) and two spatial-related posterior targets (middle occipital cortex, precuneus) derived from our fMRI (Figure 7). Analyses were carried out with MATLAB 2014b (Mathworks, Massachusetts, USA).

Functional connectivity estimation. The initial analyses estimated functional connectivity (FC) using Pearson correlations between time series (voxels within each PHG subregion) from all voxels of brain regions. All computations used Fisher's z-transformed values, which were reconverted to r-values for reporting purposes. Resting-

state fMRI time series from all other regions were used as predictors of the to-be-predicted region's resting-state fMRI time series. The resulting betas, which were directional from the predictor regions to the predicted region, were then used as FC estimates. Note that beta estimate directionality reflects optimal linear scaling of the source time series to best match the target time series (based on resting-state fMRI data).

RSFC Statistics. All statistical inferences with empirical fMRI data that produced p-values were made using two-tailed one-sample t-tests relative to 0 ($n = 430$; degrees of freedom: 429). Pearson correlation (r) was used as a measure of pattern similarity, with p-values only calculated for group-level inferences using two-sided one-sample t-tests on the Fisher's z-transformed r-values. We used a FDR correction for multiple comparisons across all calculated p-values reported in this study. This revealed an uncorrected $p < 0.004$ threshold. All p-values reported as statistically significant were below this threshold, such that all significant p-values were statistically significant ($p < 0.05$) after correcting for multiple comparisons across all analyses. For presentation purposes, only p-values below 0.00001 were reported as $p < 0.00001$ based on the convention.

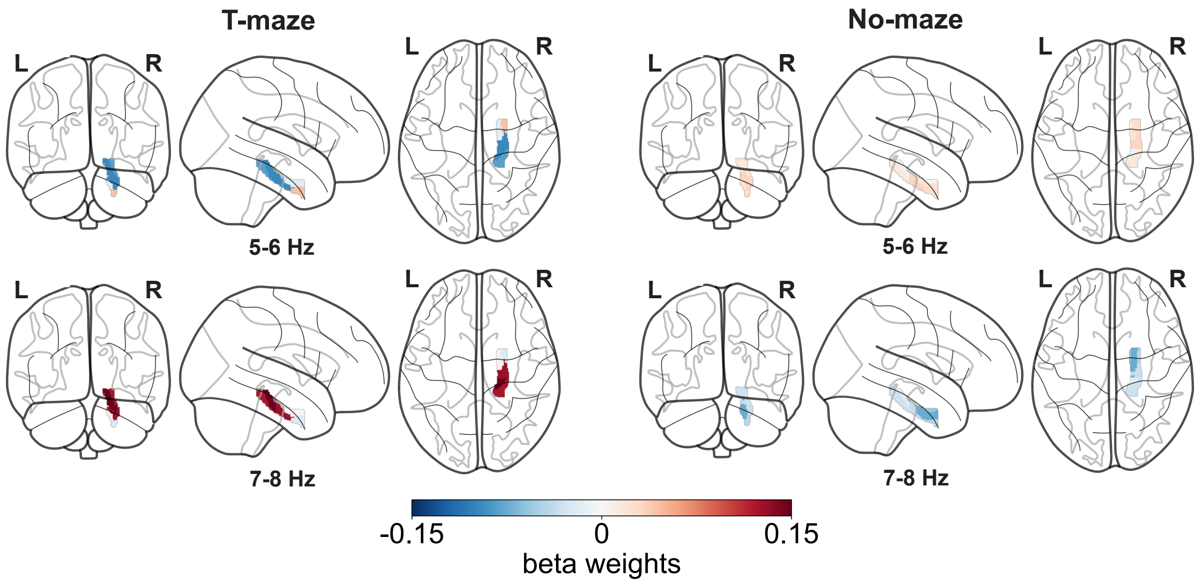

Supplementary Figure S5. **Right PHG beta weights.** Glass brain plots show the 5-6 Hz and 7-8 Hz beta weights color-coded on their respective PHG ROIs (L: left hemisphere, R: right hemisphere) separated by T-maze (left) and No-maze condition (right). Color denotes the magnitude of beta weights from the respective LME model.

Multicollinearity testing

To ensure that the EEG regressors used in the multimodal LME models presented in the results section “Single-trial right PHG activation is predicted by RPT power” were not distorted due to being intercorrelated, we investigated their variance inflation factor (VIF) (Fox & Monette, 1992). The VIF reflects how much of the variance of a regression coefficient increases due to the intercorrelation of all predictors in the model. As a general norm, VIF values should not exceed 5, since this would indicate that the standard error of the predictor in the current model is more than double compared to a model without intercorrelated predictors (McNamara et al., 2001; Thompson et al., 2017). The VIF was calculated for each EEG regressor in the LME models run for each of the six PHC ROI, in each hemisphere, and for each condition (T-maze, No-maze) by using the car package in R (Fox & Weisberg, 2018). The resulting VIF values separated by EEG regressor are depicted in Supplementary Figure S6. Notably, the largest VIF found for any regressor across all models was 2.06, which indicates at most a mild impact of correlations between regressors on the regression coefficients. Moreover, the significant regressors (5-6 Hz and 7-8 Hz) from our combined EEG-fMRI analysis were in a lower range between 1.23 and 1.63. Hence, it is safe to assume that our results were at most mildly impacted by multicollinearity.

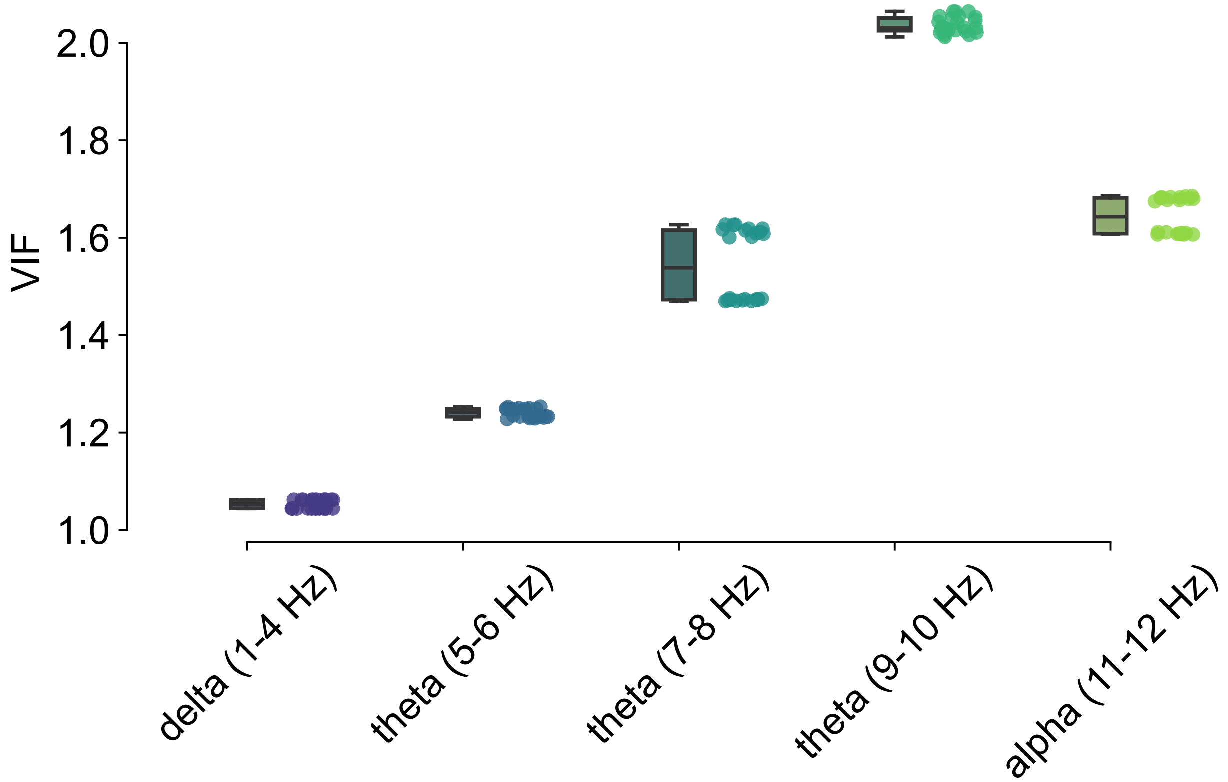

Supplementary Figure S6. **VIF across regressors.** VIF for EEG regressors (x-axis) included in LME models. Shown are individual VIF values as dots and a corresponding boxplot. Each dot represents the VIF value for the respective regressor in one of the LME models predicting PHG activation discussed in this study.

Left PHG, T-maze condition

	**aPHG1**			**aPHG2**			**aPHG3**			**aPHG4**			**pPHG1**			**pPHG2**		
Predictors	*Beta*	*t*	*p*	*Beta*	*t*	*p*	*Beta*	*t*	*p*	*Beta*	*t*	*p*	*Beta*	*t*	*p*	*Beta*	*t*	*p*
1-4 Hz	0.016 (-0.046 – 0.079)	0.513	0.729	0.030 (-0.032 – 0.092)	0.946	0.516	0.011 (-0.051 – 0.074)	0.359	0.919	0.032 (-0.029 – 0.093)	1.032	0.542	0.047 (-0.014 – 0.108)	1.508	0.396	0.038 (-0.023 – 0.099)	1.213	0.575
5-6 Hz	-0.075 (-0.151 – 0.001)	-1.938	0.159	-0.062 (-0.138 – 0.014)	-1.611	0.323	-0.012 (-0.089 – 0.064)	-0.319	0.919	-0.102 (-0.177 – -0.028)	-2.687	**0.036**	-0.074 (-0.148 – 0.001)	-1.942	0.314	-0.052 (-0.127 – 0.023)	-1.362	0.575
7-8 Hz	0.059 (-0.032 – 0.151)	1.272	0.407	0.050 (-0.042 – 0.141)	1.070	0.516	0.005 (-0.087 – 0.096)	0.102	0.919	0.116 (0.026 – 0.206)	2.520	**0.036**	0.058 (-0.032 – 0.148)	1.255	0.420	0.042 (-0.048 – 0.133)	0.923	0.575
9-10 Hz	-0.048 (-0.144 – 0.048)	-0.979	0.492	-0.017 (-0.113 – 0.079)	-0.356	0.866	0.008 (-0.088 – 0.105)	0.172	0.919	-0.044 (-0.139 – 0.051)	-0.913	0.542	0.003 (-0.092 – 0.097)	0.054	0.957	0.028 (-0.067 – 0.123)	0.579	0.575
11-12 Hz	-0.008 (-0.088 – 0.072)	-0.191	0.848	-0.001 (-0.081 – 0.079)	-0.025	0.980	-0.021 (-0.101 – 0.059)	-0.511	0.919	0.000 (-0.079 – 0.079)	0.006	0.995	-0.034 (-0.113 – 0.045)	-0.848	0.534	-0.025 (-0.104 – 0.054)	-0.628	0.575

Supplementary Table S1. Fixed Effects table of the LME model run on single-trial left PHG ROI regression weights (columns) recorded during the T-maze condition. aPHG4 BOLD was significantly related to trial-by-trial variation in RPT. Increases in 5-6 Hz and 11-12 Hz power were associated with decreases in aPHG4 activation, while 7-8 Hz power was coupled to increases in aPHG4 activation.

Right PHG, No-maze condition

	aPHG1			aPHG2			aPHG3			aPHG4			pPHG1			pPHG2		
Predictors	*Beta*	*t*	*p*	*Beta*	*t*	*p*	*Beta*	*t*	*p*	*Beta*	*t*	*p*	*Beta*	*t*	*p*	*Beta*	*t*	*p*
1-4 Hz	-0.035 (-0.093 – 0.024)	-1.168	0.365	-0.066 (-0.125 – -0.008)	-2.215	0.162	-0.053 (-0.113 – 0.006)	-1.751	0.481	-0.071 (-0.131 – -0.011)	-2.330	0.120	-0.042 (-0.102 – 0.017)	-1.392	0.487	-0.032 (-0.092 – 0.028)	-1.052	0.904
5-6 Hz	-0.002 (-0.066 – 0.062)	-0.050	0.960	0.032 (-0.033 – 0.096)	0.966	0.517	0.026 (-0.039 – 0.091)	0.779	0.873	0.021 (-0.044 – 0.087)	0.640	0.778	0.016 (-0.050 – 0.081)	0.467	0.769	-0.004 (-0.070 – 0.062)	-0.120	0.904
7-8 Hz	-0.056 (-0.128 – 0.015)	-1.540	0.365	-0.035 (-0.107 – 0.037)	-0.945	0.517	-0.042 (-0.115 – 0.031)	-1.120	0.789	-0.075 (-0.148 – -0.002)	-2.003	0.136	-0.031 (-0.105 – 0.042)	-0.835	0.606	-0.014 (-0.087 – 0.060)	-0.372	0.904
9-10 Hz	-0.059 (-0.140 – 0.022)	-1.419	0.365	-0.042 (-0.124 – 0.039)	-1.021	0.517	0.004 (-0.079 – 0.087)	0.103	0.932	-0.012 (-0.095 – 0.071)	-0.282	0.778	-0.049 (-0.132 – 0.034)	-1.167	0.487	-0.039 (-0.123 – 0.044)	-0.930	0.904
11-12 Hz	0.038 (-0.035 – 0.112)	1.023	0.368	0.011 (-0.063 – 0.085)	0.294	0.829	-0.014 (-0.089 – 0.061)	-0.377	0.932	-0.015 (-0.090 – 0.060)	-0.392	0.778	0.001 (-0.074 – 0.076)	0.035	0.972	0.022 (-0.053 – 0.097)	0.569	0.904

Supplementary Table S2. Fixed Effects table of the LME models run on single-trial BOLD from the right PHG ROIs (columns) recorded during the No-maze condition.

Left PHG, No-maze condition

	aPHG1			aPHG2			aPHG3			aPHG4			pPHG1			pPHG2		
Predictors	*Beta*	*t*	*p*	*Beta*	*t*	*p*	*Beta*	*t*	*p*	*Beta*	*t*	*p*	*Beta*	*t*	*p*	*Beta*	*t*	*p*
1-4 Hz	-0.059 (-0.118 – 0.001)	-1.935	0.160	-0.059 (-0.118 – 0.001)	-1.940	0.158	0.009 (-0.052 – 0.071)	0.298	0.789	-0.042 (-0.102 – 0.017)	-1.404	0.558	-0.036 (-0.095 – 0.024)	-1.184	0.422	-0.054 (-0.113 – 0.006)	-1.771	0.461
5-6 Hz	-0.017 (-0.083 – 0.048)	-0.519	0.850	0.003 (-0.062 – 0.068)	0.084	0.933	0.025 (-0.043 – 0.092)	0.715	0.789	0.013 (-0.051 – 0.078)	0.403	0.687	0.034 (-0.031 – 0.100)	1.039	0.422	0.036 (-0.029 – 0.102)	1.094	0.548
7-8 Hz	-0.065 (-0.138 – 0.008)	-1.741	0.164	-0.076 (-0.149 – -0.004)	-2.060	0.158	-0.056 (-0.131 – 0.019)	-1.471	0.789	-0.046 (-0.118 – 0.027)	-1.232	0.558	-0.040 (-0.113 – 0.033)	-1.077	0.422	-0.048 (-0.121 – 0.025)	-1.286	0.548
9-10 Hz	-0.016 (-0.098 – 0.067)	-0.374	0.850	-0.016 (-0.098 – 0.067)	-0.374	0.933	0.017 (-0.068 – 0.101)	0.383	0.789	-0.042 (-0.124 – 0.041)	-0.993	0.558	-0.039 (-0.122 – 0.043)	-0.931	0.422	-0.013 (-0.095 – 0.070)	-0.304	0.913
11-12 Hz	0.007 (-0.068 – 0.081)	0.177	0.859	0.008 (-0.067 – 0.082)	0.200	0.933	-0.010 (-0.087 – 0.066)	-0.268	0.789	0.034 (-0.040 – 0.108)	0.893	0.558	-0.005 (-0.079 – 0.070)	-0.129	0.898	0.002 (-0.072 – 0.077)	0.064	0.949

Supplementary Table S3. Fixed Effects table of the LME models run on single-trial BOLD from the left PHG ROIs (columns) recorded during the No-maze condition.

Control ROIs, T-maze condition

	Left Heschl's Gyrus			Right Heschl's Gyrus			Precuneus		
Predictors	*Estimates*	*CI*	*p*	*Estimates*	*CI*	*p*	*Estimates*	*CI*	*p*
1-4 Hz	-0.015	-0.069 – 0.038	0.924	-0.019	-0.071 – 0.033	0.716	-0.004	-0.084 – 0.075	0.916
5-6 Hz	-0.001	-0.046 – 0.044	0.950	-0.005	-0.048 – 0.039	0.833	-0.041	-0.107 – 0.025	0.446
7-8 Hz	0.010	-0.030 – 0.050	0.924	0.028	-0.011 – 0.067	0.458	0.027	-0.032 – 0.086	0.560
9-10 Hz	-0.003	-0.052 – 0.047	0.950	-0.008	-0.056 – 0.040	0.833	0.055	-0.017 – 0.128	0.400
11-12 Hz	-0.013	-0.059 – 0.033	0.924	-0.018	-0.062 – 0.027	0.716	-0.085	-0.152 – -0.018	0.081

Supplementary Table S4. Fixed Effects table of the LME models run on single-trial BOLD from the control ROIs (left and right Heschl's Gyrus, Precuneus; columns) recorded during the T-maze condition.

Parietal-temporal Glassor ROIs, T-maze condition

	Parahippocampal Area 3 (right)			Perirhinal Ectorhinal Cortex (right)			Ventral Intraparietal Complex (left)		
Predictors	*Beta*	*t*	*p*	*Beta*	*t*	*p*	*Beta*	*t*	*p*
1-4 Hz	-0.006 (-0.068 – 0.055)	-0.205	0.837	-0.057 (-0.118 – 0.004)	-1.848	0.130	0.018 (-0.043 – 0.078)	0.567	0.685
5-6 Hz	-0.097 (-0.172 – -0.022)	-2.543	**0.045**	0.109 (0.035 – 0.183)	2.898	**0.023**	-0.101 (-0.176 – -0.027)	-2.671	**0.046**
7-8 Hz	0.112 (0.022 – 0.203)	2.435	**0.045**	0.098 (0.008 – 0.187)	2.149	0.096	0.076 (-0.014 – 0.166)	1.656	0.196
9-10 Hz	-0.051 (-0.146 – 0.044)	-1.052	0.439	0.008 (-0.086 – 0.102)	0.171	0.865	-0.032 (-0.126 – 0.062)	-0.665	0.685
11-12 Hz	-0.013 (-0.092 – 0.066)	-0.315	0.837	-0.027 (-0.105 – 0.052)	-0.667	0.606	0.000 (-0.078 – 0.079)	0.006	0.995

Supplementary Table S5. Fixed Effects table of the LME models run on single-trial BOLD from the Glasser atlas ROIs (columns) recorded during the T-maze condition. Only the parietal-temporal ROIs with at least one significant beta weight are shown.

Frontal Glassor ROIs, T-maze condition

	Anterior Area 24' (left)			Area 33' (left)			Dorsal Area 8A (left)			Ventral Area 8A (left)		
Predictors	*Beta*	*t*	*p*	*Beta*	*t*	*p*	*Beta*	*t*	*p*	*Beta*	*t*	*p*
1-4 Hz	0.029 (-0.032 – 0.090)	0.935	0.420	0.024 (-0.034 – 0.082)	0.818	0.620	0.011 (-0.050 – 0.072)	0.359	0.841	0.016 (-0.045 – 0.077)	0.519	0.604
5-6 Hz	-0.131 (-0.206 – -0.057)	-3.471	**0.003**	-0.089 (-0.160 – -0.018)	-2.449	**0.044**	-0.095 (-0.170 – -0.020)	-2.500	**0.025**	-0.131 (-0.205 – -0.057)	-3.457	**0.002**
7-8 Hz	0.151 (0.061 – 0.241)	3.295	**0.003**	0.112 (0.026 – 0.198)	2.556	**0.044**	0.120 (0.030 – 0.210)	2.609	**0.025**	0.179 (0.090 – 0.269)	3.920	**0.001**
9-10 Hz	-0.065 (-0.159 – 0.029)	-1.358	0.262	-0.040 (-0.130 – 0.050)	-0.871	0.620	-0.059 (-0.154 – 0.035)	-1.228	0.330	-0.081 (-0.175 – 0.013)	-1.683	0.139
11-12 Hz	-0.004 (-0.082 – 0.075)	-0.094	0.925	0.017 (-0.058 – 0.092)	0.453	0.706	0.008 (-0.071 – 0.087)	0.200	0.841	0.023 (-0.056 – 0.101)	0.570	0.604

	Anterior Area IFJ (right)			Posterior Area IFJ (right)			Dorsal Area 32 (left)			Posterior Area 32' (left)		
Predictors	*Beta*	*t*	*p*	*Beta*	*t*	*p*	*Beta*	*t*	*p*	*Beta*	*t*	*p*
1-4 Hz	0.027 (-0.031 – 0.085)	0.916	0.432	0.007 (-0.051 – 0.065)	0.225	0.840	0.010 (-0.050 – 0.069)	0.323	0.747	0.012 (-0.049 – 0.072)	0.375	0.849
5-6 Hz	-0.114 (-0.185 – -0.043)	-3.139	**0.010**	-0.069 (-0.140 – 0.002)	-1.913	0.112	-0.090 (-0.163 – -0.018)	-2.436	**0.045**	-0.127 (-0.201 – -0.052)	-3.350	**0.005**
7-8 Hz	0.129 (0.043 – 0.215)	2.937	**0.010**	0.136 (0.050 – 0.222)	3.108	**0.012**	0.119 (0.031 – 0.207)	2.651	**0.045**	0.131 (0.041 – 0.221)	2.871	**0.013**
9-10 Hz	-0.067 (-0.158 – 0.023)	-1.470	0.213	-0.064 (-0.154 – 0.026)	-1.402	0.242	-0.074 (-0.166 – 0.018)	-1.577	0.230	-0.004 (-0.097 – 0.090)	-0.074	0.941
11-12 Hz	-0.020 (-0.095 – 0.055)	-0.533	0.594	0.008 (-0.067 – 0.083)	0.201	0.840	0.013 (-0.063 – 0.090)	0.345	0.747	-0.029 (-0.108 – 0.049)	-0.737	0.849

Supplementary Table S6. Fixed Effects table of the LME models run on single-trial BOLD from the Glasser atlas ROIs (columns) recorded during the T-maze condition. Only the frontal ROIs with at least one significant beta weight are shown.

Experiment	Modality	Task	Sample size	Main outcome measures	Analysis	Main findings (RPT effect)	Observed over left hemisphere?	RPT theta range	Peak RPT channel	Main conclusion
**Baker & Holroyd, 2009**										
1	EEG	Virtual T-maze task	12	ERP amplitude and ERP peak latency	2-way repeated measures ANOVA	7 ms ERP peak latency difference (left > right), no ERP amplitude difference	No		PO8	First established RPT effect using ERPs
2	EEG	Tuning fork shaped maze task (TF-maze)	12	ERP amplitude and ERP peak latency	3-way repeated measures ANOVA	10 ms ERP peak latency difference (lef > right), ERP amplitude difference (right > left)	No		PO8	First established RPT effect using ERPs
**Baker & Holroyd, 2013**										
1	EEG	Maze/No-maze task	12	ERP amplitude and ERP peak latency	3-way repeated measures ANOVA	8 ms ERP peak latency difference (left > right) only for T-maze condition, ERP amplitude difference (right > left) only for T-maze condition	No		PO8	RPT effect is specific to spatial navigation
2	EEG	Virtual T-maze task	91	ERP amplitude and ERP peak latency	2-way repeated measures ANOVA	6 ms ERP peak latency difference (left > right), ERP amplitude difference (right > left)	No		PO8	Successful replication of RPT effect
				Source localization solution	EEG source analysis (equivalent current dipole model)	Source analysis of ERP indicated bilateral PHG	No		PO8	RPT can be source localized to PHG
				RPT power and RPT peak latency	2-way repeated measures ANOVA	RPT power (right > left) and 20 ms RPT peak latency difference (left > right)	No	7-10 Hz	PO8	First established RPT effect using time-frequency analysis

				ITC and ITC peak latency	Paired t-test	ITC difference (right > left) and 14 ms ITC peak latency difference (left > right)	No	7-10 Hz	PO8	RPT is consistent with a partial phase reset
				Single-trial phase alignment (RVL)	Harrison-Kanji test	RVL difference (post > pre and right > left)	No	7-10 Hz	PO8	RPT is consistent with a partial phase reset
3	EEG	Complex T-maze task (CT-maze)	37	ERP amplitude and ERP peak latency	3-way repeated measures ANOVA	9 ms ERP peak latency difference (left > right), no ERP amplitude difference	No		PO8	Successful replication of RPT effect
				Reconstruction (i.e., drawing) of CT-maze	Levene's Test for Equality of Variances, paired t-test	ERP peak latency difference (left > right, 10 ms) for subjects who successfully recalled the CT-maze's shape than for those who did not (4 ms)	No			RPT effect is related to spatial memory
**Baker et al., 2015**										
1	fMRI	Maze/No-maze task	16	Whole brain clusters (T-maze > No-maze)	General linear model, paired t-test	Significant clusters in bilateral HC, PHG, MTC and PC (T-maze > No-maze)	No			HC and PHG activation underlie navigation in T-maze
				ROI contrast in right PHG (right > left alley only in T-maze and only in No-Maze)	General linear model, paired t-test	Larger PHG activation in posterior and anterior ROIs (right > left, only for T-maze)	No			PHG activation follows RPT effect pattern
**Lin et al., 2022**										
1	EEG & virtual reality	Virtual reality adaptation of T-maze task	22	RPT power and RPT peak latency	2-way repeated measures ANOVA	No RPT power or RPT peak latency difference	No	6-8 Hz	P8	RPT power is visible in real-world navigation
				Movement-related RPT	Cluster-based permutation test	Theta power increased with movement speed after junction point	Yes	5-7 Hz	P3	Theta power during T-maze navigation is sensitive to movement speed
**Current study**										
1	EEG	Virtual T-maze task	11	RPT power and RPT peak latency	Wilcoxon signed-rank test	RPT power (right > left) and 16 ms RPT peak latency difference (left > right)	No	7-10 Hz	PO8	Successful replication of RPT effect
				ITC and ITC peak latency	Wilcoxon signed-rank test	ITC difference (right > left) and 14 ms ITC peak latency difference (left > right)	No	7-10 Hz	PO8	Successful replication of evidence for partial phase reset
				Single-trial phase alignment (RVL, pre- vs. post-feedback, left vs. right)	Harrison-Kanji test, Watson-Williams test	RVL difference (post > pre), but not for alley	No	7-10 Hz	PO8	Partial replication of evidence for partial phase reset
1	MEG	Virtual T-maze task	11	RPT power and RPT peak latency	Wilcoxon signed-rank test	RPT power (right-left) and 28 ms RPT peak latency difference (left-right)	No	7-10 Hz	MRO33	RPT is not a result of smearing through volume conduction
				ITC and ITC peak latency	Wilcoxon signed-rank test	ITC difference (right > left), but no ITC peak latency difference	No	7-10 Hz	MRO33	Partial replication of evidence for partial phase reset
				Single-trial phase alignment (RVL)	Harrison-Kanji test, Watson-Williams test	RVL difference (post > pre and right > left)	No	7-10 Hz	MRO33	Successful replication of evidence for partial phase reset
2	Simultaneous EEG-fMRI	Maze/No-maze task	25	RPT power and RPT peak latency	2-way repeated measures ANOVA	29 ms RPT peak latency difference (left > right), no RPT power difference	No	7-10 Hz	E160 (PO8)	Partial replication of RPT effect with concurrent fMRI
				Whole brain clusters (T-maze > No-maze)	General linear model, paired t-test	Significant clusters in bilateral HC, PHG, MTC and PC (T-maze > No-maze)	No			Successful replication of fMRI results
				ROI contrast in right PHG (right > left alley only in T-maze and only in No-Maze)	General linear model, paired t-test	Larger PHG activation in posterior and anterior ROIs (right > left, only for T-maze)	No			Successful replication of fMRI results
				Single-trial coupling of RPT power and PHG BOLD activation	LME models predicting PHG activation with RPT	RPT regressors significantly predicted single-trial PHG activation in two posterior and one anterior ROI	Yes, for left hemisphere ROI (APHG4) and sensor (E107)	5-6 Hz, 7-8 Hz	E160 (PO8)	RPT is at least partially generated by PHG activation

Supplementary Table S7. Overview of our previous work on the RPT effect using the T-maze task and replication efforts. Columns are details of individual studies (e.g., modality, task, main findings, etc). Rows are methods, findings, and conclusions nested within experiments and studies.

References

Behrens, T. E. J., Johansen-Berg, H., Woolrich, M. W., Smith, S. M., Wheeler-Kingshott, C. a. M., Boulby, P. A., Barker, G. J., Sillery, E. L., Sheehan, K., Ciccarelli, O., Thompson, A. J., Brady, J. M., & Matthews, P. M. (2003). Non-invasive mapping of connections between human thalamus and cortex using diffusion imaging. *Nature Neuroscience*, *6*(7), Article 7. https://doi.org/10.1038/nn1075

Cohen, M. X., Schoene-Bake, J.-C., Elger, C. E., & Weber, B. (2009). Connectivity-based segregation of the human striatum predicts personality characteristics. *Nature Neuroscience*, *12*(1), Article 1. https://doi.org/10.1038/nn.2228

Fan, L., Li, H., Zhuo, J., Zhang, Y., Wang, J., Chen, L., Yang, Z., Chu, C., Xie, S., Laird, A. R., Fox, P. T., Eickhoff, S. B., Yu, C., & Jiang, T. (2016). The Human Brainnetome Atlas: A New Brain Atlas Based on Connectional Architecture. *Cerebral Cortex*, *26*(8), 3508–3526. https://doi.org/10.1093/cercor/bhw157

Fan, L., Wang, J., Zhang, Y., Han, W., Yu, C., & Jiang, T. (2014). Connectivity-Based Parcellation of the Human Temporal Pole Using Diffusion Tensor Imaging. *Cerebral Cortex*, *24*(12), 3365–3378. https://doi.org/10.1093/cercor/bht196

Fox, J., & Monette, G. (1992). Generalized Collinearity Diagnostics. *Journal of the American Statistical Association*, *87*(417), 178–183. https://doi.org/10.1080/01621459.1992.10475190

Fox, J., & Weisberg, S. (2018). *An R companion to applied regression*. Sage publications. https://books.google.com/books?hl=en&lr=&id=SfNrDwAAQBAJ&oi=fnd&pg=PP13&dq=Fox+J,+Weisberg+S+(2019).+_An+R+Companion+to+Applied+Regression_,+Third+edition.+Sage,+Thousand+Oaks+CA.+%3Chttps://www.john-fox.ca/Companion/%3E.&ots=EqKjb9epWu&sig=o8iz675QGJdRanfm6eaQDuvwyfc

Glasser, M. F., Sotiropoulos, S. N., Wilson, J. A., Coalson, T. S., Fischl, B., Andersson, J. L., Xu, J., Jbabdi, S., Webster, M., Polimeni, J. R., Van Essen, D. C., & Jenkinson, M. (2013). The minimal preprocessing pipelines for the Human

Connectome Project. *NeuroImage*, *80*, 105–124. https://doi.org/10.1016/j.neuroimage.2013.04.127

Huntgeburth, S. C., & Petrides, M. (2012). Morphological patterns of the collateral sulcus in the human brain. *European Journal of Neuroscience*, *35*(8), 1295–1311. https://doi.org/10.1111/j.1460-9568.2012.08031.x

Jbabdi, S., Sotiropoulos, S. N., Savio, A. M., Graña, M., & Behrens, T. E. J. (2012). Model-based analysis of multishell diffusion MR data for tractography: How to get over fitting problems. *Magnetic Resonance in Medicine*, *68*(6), 1846–1855. https://doi.org/10.1002/mrm.24204

Johansen-Berg, H., Behrens, T. E. J., Robson, M. D., Drobnjak, I., Rushworth, M. F. S., Brady, J. M., Smith, S. M., Higham, D. J., & Matthews, P. M. (2004). Changes in connectivity profiles define functionally distinct regions in human medial frontal cortex. *Proceedings of the National Academy of Sciences*, *101*(36), 13335–13340. https://doi.org/10.1073/pnas.0403743101

Liu, H., Qin, W., Li, W., Fan, L., Wang, J., Jiang, T., & Yu, C. (2013). Connectivity-Based Parcellation of the Human Frontal Pole with Diffusion Tensor Imaging. *Journal of Neuroscience*, *33*(16), 6782–6790. https://doi.org/10.1523/JNEUROSCI.4882-12.2013

MacDonald, D. (1996). MNI-display: Program for display and segmentation of surfaces and volumes. *McConnel Brain Imaging Center, Montreal Neurological Institute*. http://www.bic.mni.mcgill.ca/~stever/Software/RelNotes/Display.ps

McNamara, B., Ray, J. L., Arthurs, O. J., & Boniface, S. (2001). Transcranial magnetic stimulation for depression and other psychiatric disorders. *Psychological Medicine*, *31*(7), 1141–1146. https://doi.org/10.1017/S0033291701004378

Saygin, Z. M., Osher, D. E., Augustinack, J., Fischl, B., & Gabrieli, J. D. E. (2011). Connectivity-based segmentation of human amygdala nuclei using probabilistic tractography. *NeuroImage*, *56*(3), 1353–1361. https://doi.org/10.1016/j.neuroimage.2011.03.006

Shi, J., & Malik, J. (2000). Normalized cuts and image segmentation. *IEEE Transactions on Pattern Analysis and Machine Intelligence*, *22*(8), 888–905. https://doi.org/10.1109/34.868688

Sotiropoulos, S. N., Jbabdi, S., Xu, J., Andersson, J. L., Moeller, S., Auerbach, E. J., Glasser, M. F., Hernandez, M., Sapiro, G., Jenkinson, M., Feinberg, D. A., Yacoub, E., Lenglet, C., Van Essen, D. C., Ugurbil, K., & Behrens, T. E. J. (2013). Advances in diffusion MRI acquisition and processing in the Human Connectome Project. *NeuroImage*, *80*, 125–143. https://doi.org/10.1016/j.neuroimage.2013.05.057

Thompson, C. G., Kim, R. S., Aloe, A. M., & Becker, B. J. (2017). Extracting the Variance Inflation Factor and Other Multicollinearity Diagnostics from Typical Regression Results. *Basic and Applied Social Psychology*, *39*(2), 81–90. https://doi.org/10.1080/01973533.2016.1277529

Van Essen, D. C., Ugurbil, K., Auerbach, E., Barch, D., Behrens, T. E. J., Bucholz, R., Chang, A., Chen, L., Corbetta, M., Curtiss, S. W., Della Penna, S., Feinberg, D., Glasser, M. F., Harel, N., Heath, A. C., Larson-Prior, L., Marcus, D., Michalareas, G., Moeller, S., … Yacoub, E. (2012). The Human Connectome Project: A data acquisition perspective. *NeuroImage*, *62*(4), 2222–2231. https://doi.org/10.1016/j.neuroimage.2012.02.018

Zhang, F., Kahali, P., Suter, Y., Norton, I., Rigolo, L., Savadjiev, P., Song, Y., Rathi, Y., Cai, W., Wells, W. M., Golby, A. J., & O'Donnell, L. J. (2017). Automated connectivity-based groupwise cortical atlas generation: Application to data of neurosurgical patients with brain tumors for cortical parcellation prediction. *2017 IEEE 14th International Symposium on Biomedical Imaging (ISBI 2017)*, 774–777. https://doi.org/10.1109/ISBI.2017.7950633

Zhang, P. (2015). Evaluating accuracy of community detection using the relative normalized mutual information. *Journal of Statistical Mechanics: Theory and Experiment*, *2015*(11), P11006. https://doi.org/10.1088/1742-5468/2015/11/P11006

Zhang, Y., Larcher, K. M.-H., Misic, B., & Dagher, A. (2017). Anatomical and functional organization of the human substantia nigra and its connections. *eLife*, *6*, e26653. https://doi.org/10.7554/eLife.26653
